# FerroScore: a statistical approach for quantifying tumor-related ferroptosis based on omics data

**DOI:** 10.1093/bib/bbag368

**Published:** 2026-07-06

**Authors:** Jiaqi Teng, Qi Gong, Zhaohang Cai, Tianshou Zhou

**Affiliations:** School of Mathematics, Sun Yat-sen University, No. 135 Xingang Xi Road, Haizhu District, Guangzhou, Guangdong Province 510275, China; School of Mathematics, Sun Yat-sen University, No. 135 Xingang Xi Road, Haizhu District, Guangzhou, Guangdong Province 510275, China; School of Mathematics, Sun Yat-sen University, No. 135 Xingang Xi Road, Haizhu District, Guangzhou, Guangdong Province 510275, China; School of Mathematics, Sun Yat-sen University, No. 135 Xingang Xi Road, Haizhu District, Guangzhou, Guangdong Province 510275, China

**Keywords:** ferroptosis, tumor, single-cell RNA sequencing, network, computational biology

## Abstract

Ferroptosis is a novel form of programmed cell death driven by iron-dependent lipid peroxidation, and can significantly influence the progression of complex diseases such as cancer. Current methods of detecting ferroptosis rely primarily on experimental techniques that are typically low-throughput and costly, limiting their clinical applications. Here we develop an effective statistical method, FerroScore, to quantify ferroptosis by generating a score that integrates the activities of three core pathways—iron, glutathione, and lipid metabolism. This method enables the cross-resolution assessment of ferroptosis and provides mechanistic insights into tumor, immune, and neurodegenerative diseases, thus having potential applications in targeted therapy and drug discovery. When applied to pancreatic cancer transcriptomic data, FerroScore reveals: (i) a U-shaped relationship between ferroptosis and patient survival; (ii) heterogeneous ferroptosis activity across cell types in the tumor microenvironment, with high sensitivity to Macrophages, CD8 Tcm cells, and a population of nCAFs; (iii) the role of ferroptosis-active cells in reshaping the immunosuppressive and pro-metastatic microenvironment through intercellular communication.

## Introduction

Ferroptosis is characterized by iron-dependent lipid peroxidation [1] and is a signature of cell death. Unlike classical forms of cell death such as apoptosis, necrosis, and autophagy, ferroptosis exhibits unique biological features involving dysregulated iron metabolism, accumulation of reactive oxygen species (ROS), and oxidative damage to membrane lipids [2]. It contributes not only to tissue homeostasis but also plays a critical role in disease pathogenesis, particularly in cancer. Ferroptosis has been identified in multiple organ systems, including the nervous system [3], heart [4], liver [5], gastrointestinal tract [6], lung [7], kidney [8], and pancreas [9]. Targeted ferroptosis through induction or inhibition has emerged as a promising therapeutic strategy of cancers and neurodegenerative diseases, providing new directions for clinical intervention [10, 11].

Ferroptosis is regulated metabolically through three core pathways: iron, glutathione (GSH), and lipid metabolism [12]. Under physiological conditions, cellular iron homeostasis is maintained by the redox cycling between ferrous (Fe^2+^) and ferric (Fe^3+^) iron, supporting essential cellular functions [13]. However, dysregulation of iron metabolism causes excessive Fe^2+^ accumulation, which drives the Fenton reaction to generate ROS, thereby disrupting redox balance and inducing oxidative stress. Such persistent stress impairs cellular structure and function and simultaneously weakens the antioxidant defense system. When GSH and glutathione peroxidase 4 (GPX4), as key components of the system, are compromised or depleted, excessive ROS accumulation accelerates lipid peroxidation, ultimately triggering ferroptosis [14].

Current detection of ferroptosis relies predominantly on experimental assays, including intracellular iron measurement with FerroOrange [15], GSH quantification via DTNB assays [16], lipid peroxidation detection using C11-BODIPY probes [17], and cell viability assessment with CCK-8 kits [18]. Molecular techniques like qPCR and Western blotting provide complementary evidence by monitoring key regulators such as GPX4, ACSL4, and FSP1 [19]. Although informative, these methods are low throughput, resource intensive, and largely restricted to preselected markers or bulk measurements, limiting their utility for uncovering novel regulators or resolving cellular heterogeneity. Transcriptomic computational strategies have increasingly been used to infer ferroptosis activity, including gene set enrichment methods such as Single Sample Gene Set Enrichment Analysis (ssGSEA) and Gene Set Variation Analysis (GSVA), FerrDb-derived signatures, and scoring schemes like the Ferroptosis Potential Index (FPI) and Ferroptosis Score (FPS) [20–23]. These methods enable scalable estimation of ferroptosis-related transcriptional states, particularly in bulk tumor cohorts. However, most rely on predefined gene sets or single sample enrichment statistics, and thus mainly capture the average expression tendency of ferroptosis-associated genes. This design limits their ability to distinguish ferroptosis execution from compensatory stress responses and may incompletely capture the coordinated network dynamics of iron, GSH, and lipid metabolism in heterogeneous samples. Routine clinical indicators, such as circulating markers of iron, GSH, or lipid metabolism, offer another accessible but indirect source of information. However, these systemic measurements lack tissue and cell-type resolution, are susceptible to confounding by overlapping pathological processes, and remain insufficiently linked to molecular ferroptosis mechanisms.

To overcome the limitations of current ferroptosis detection methods in cost, throughput, and resolution, we developed FerroScore, a computational tool that quantifies ferroptosis activity from transcriptomic data at single-sample or single-cell resolution. By integrating expression patterns of core ferroptosis-associated genes with network topology analysis, FerroScore supports cross-resolution evaluation and enables the unveiling of regulatory mechanisms. Applied to bulk and single-cell RNA-seq (scRNA-seq) datasets of pancreatic cancer, FerroScore reveals ferroptosis features and mechanisms across tissue and cellular scales. We further investigate how ferroptosis remodels the tumor immune microenvironment, with results providing mechanistic insights into tumor-immune interactions. We also use FerroScore to analyze Alzheimer’s disease (AD) data, exploring the potential mechanisms and application prospects of ferroptosis in non-oncological contexts such as neurodegeneration. In a word, FerroScore offers an effective and interpretable framework for assessing ferroptosis activity, deciphering regulatory networks, and identifying potential therapeutic targets, providing useful information for understanding and intervening ferroptosis-related diseases.

## Methods

### Overview of FerroScore

FerroScore is an algorithm that quantifies the ferroptosis activity based on transcriptomic data (RNA-seq or scRNA-seq). It enables quantitative assessment of ferroptosis in individual samples or single cells by systematically integrating key molecular features and network topology related to ferroptosis.

**Figure 1:**
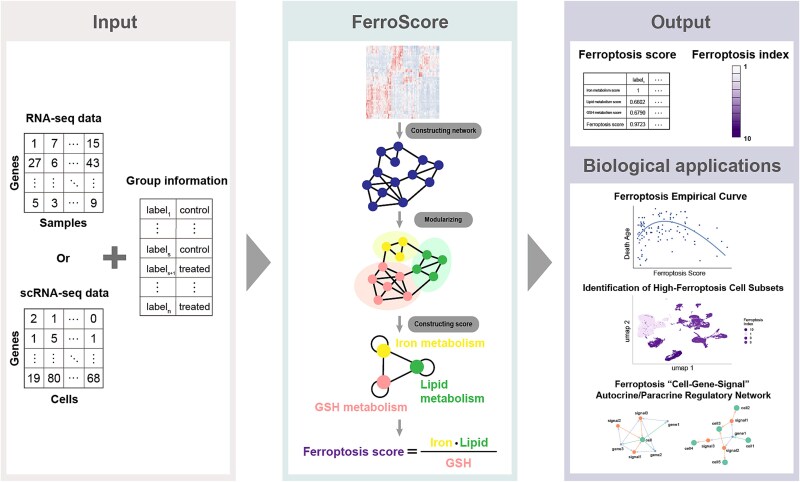
Overview of FerroScore. FerroScore converts transcriptomic data into quantitative ferroptosis metrics. The pipeline takes RNA-seq or scRNA-seq data and associated labels as input (“Input”). It constructs a PPI network from DEGs, refines it into a core ferroptosis network via module identification, functional annotation, and merging, and uses topological features to generate a continuous activity score and a stratified index (range: 1–10) (“FerroScore”). These outputs enable diverse downstream analyses, including association with clinical survival, identification of high-activity cell subpopulations, and inference of autocrine and paracrine regulatory networks (“Output”).

Specifically, FerroScore first constructs a protein–protein interaction (PPI) network from differentially expressed genes (DEGs) identified relative to a reference group. This establishes a cohort-dependent context in which individual samples are subsequently evaluated. Accordingly, FerroScore represents a group-relative metric that provides single sample or single cell resolution outputs within a defined biological context rather than a reference-free absolute measure. Further, it uses the Leiden algorithm [24] to identify network modules, which are automatically annotated through functional enrichment analysis and subsequently merged into a super-node network. Based on this structure, super-nodes associated with three core ferroptosis pathways—iron, GSH, and lipid metabolism—are extracted to form a dedicated ferroptosis network. This network is then quantitatively assessed using multiple topological metrics (e.g. modularity, clustering coefficient). Finally, activity scores of the three core pathways are integrated to generate a continuous ferroptosis score, accompanied by a stratified ferroptosis index ranging from 1 to 10. Based on these outcomes, FerroScore supports diverse downstream analyses. For instance, at the individual level, it assesses associations between ferroptosis activity and clinical outcomes such as survival. At the cellular level, it identifies subpopulations with high ferroptosis activity and characterizes their autocrine and paracrine regulatory networks (Fig. 1). The algorithm detects key regulatory modules and pathways, providing interpretable insights into ferroptosis mechanisms. Moreover, FerroScore is compatible with multi-resolution transcriptomic data and robustly handles cellular heterogeneity, thus being a versatile computational tool for investigating ferroptosis in disease mechanisms and therapeutic development.

## Results and discussion

### Benchmarking of FerroScore on experimental datasets

To validate FerroScore, we analyzed two publicly available experimental datasets: a mouse MEF cell line from “Molecular Cell” (2023) [25] and a human A549 cell line from “Nature Communications” (2024) [26]. Both datasets provided raw sequencing data, treatment information, and experimentally measured ferroptosis phenotypes, thereby serving as reliable benchmarks for algorithm evaluation.

**Figure 2:**
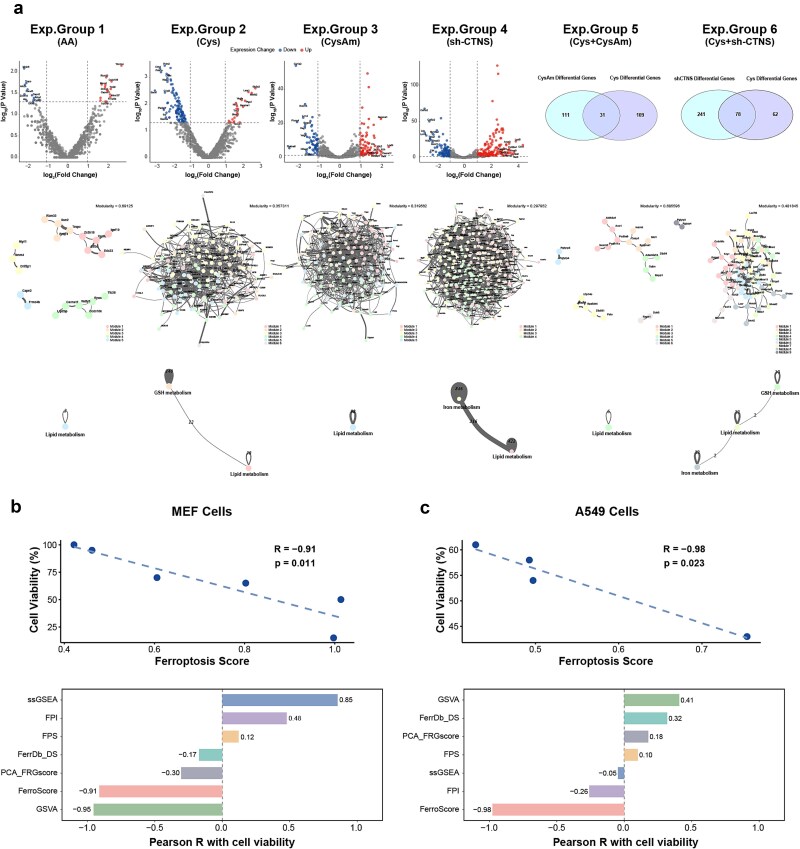
Validation of FerroScore on experimental datasets. (a) Key analytical outputs for the MEF cell line under six experimental groups (AA, Cys, CysAm, sh-CTNS, Cys + CysAm, and Cys + sh-CTNS). Panels include: a volcano plot of DEGs (distinct dot groups denote significantly up- and down-regulated genes, respectively); a Venn diagram; a modular PPI network; and the extracted ferroptosis network (edge thickness reflects connection strength). (b–c) Comprehensive benchmarking of FerroScore against existing ferroptosis scoring methods in (b) MEF cells and (c) A549 cells. In each panel, the top section shows the correlation between FerroScore and experimentally measured cell viability, with dashed lines indicating linear regression fits. The bottom section shows the Pearson correlation coefficient ranking comparing FerroScore with ssGSEA, GSVA, FPI, FPS, FerrDb_DS, and PCA_FRGscore. Each point in the scatter plots represents an experimental group.

For the mouse MEF cell line, FerroScore was applied to six experimental conditions, ranging from single amino acid starvation to combinatorial treatments (Figs 2a, S1). We benchmarked FerroScore against ferroptosis-related scoring methods, including FPI, FPS, the FerrDb-derived driver suppressor score (FerrDb_DS), the PCA-based ferroptosis-related gene score (PCA_FRGscore), and conventional gene set enrichment methods, including ssGSEA and GSVA. FerroScore exhibited a robust negative correlation with cell viability across all conditions ($R=-0.91,\,\, P=.011$), reflecting that higher scores correspond to increased cell death (Fig. 2b, top). In the multi-method comparison, FerroScore maintained a strong phenotype concordant association and retained the ability to evaluate combinatorial perturbations such as Cys + CysAm and Cys + sh-CTNS (Figs 2b, bottom; S3A). This capability arises from its network-based intersection strategy, which extracts a conserved functional core across independent perturbations rather than requiring matched transcriptome data for every combined condition. Beyond quantitative accuracy, FerroScore revealed context specific pathway alterations, such as endoplasmic reticulum stress and one-carbon folate metabolism, implicating ATF4 in lysosomal cystine regulation, consistent with the original study.

To further assess cross species applicability, we evaluated the same benchmark in the human A549 cell line treated with IKE or RSL3, each with and without PAOX/SMOX double knockout (Fig. S2). FerroScore again showed a strong negative correlation with cell viability ($R=-0.98,\,\, P=.023$) (Fig. 2c, top). By contrast, conventional enrichment and signature-based methods showed weaker or less consistent associations across the two datasets. For example, GSVA captured a negative trend in MEF cells but did not maintain comparable performance in A549 cells, while FPI and FPS showed context dependent associations under these perturbation settings (Figs 2c, bottom; S3B).

This performance difference reflects a methodological distinction. Conventional enrichment and signature-based methods mainly summarize expression changes of predefined ferroptosis gene sets, whereas FerroScore maps differential expression onto the PPI network and integrates topological features of functionally coherent modules. By prioritizing functional hubs, module coupling, and the coordinated behavior of iron, GSH, and lipid metabolism, FerroScore quantifies the network state of ferroptosis rather than simple expression intensity. This network-aware design helps reduce transcriptional noise and supports consistent phenotype concordance. Moreover, FerroScore performs consistently across different PPI network sizes ($n\le 50\,\, $to$n\ge 300$), further demonstrating its generalizability.

### Sensitivity and robustness analysis of FerroScore

To verify that the predictive performance of the FerroScore arises from its network-topological design rather than parameter-specific tuning, we conducted systematic sensitivity analyses across four methodological dimensions: expression filtering, PPI network construction, metric scaling, and reference background selection.

For expression filtering, we compared the original stringent threshold (CPM > 100) with a commonly used relaxed cutoff (CPM > 1). As detailed in the ***SI*** (Figs S4–S5, S6A), the inclusion of low-abundance genes under the relaxed threshold substantially altered the network topology and super-node composition, resulting in disrupted FerroScore values and loss of phenotypic correlation in both the MEF and A549 datasets. These results indicate that FerroScore primarily captures ferroptosis activity driven by robustly expressed core metabolic regulators, supporting the use of stringent filtering to suppress transcriptional noise and preserve biologically coherent network structure.

To assess sensitivity to PPI network construction, we evaluated alternative STRING confidence thresholds (300 and 500) in addition to the default cutoff of 400 (Fig. S6B). Across both datasets, varying the PPI threshold did not affect network topology, module organization, or super-node structure, and FerroScore values remained identical under all tested conditions. This stability demonstrates that FerroScore is insensitive to moderate variations in PPI confidence filtering and is governed by a conserved core ferroptosis interaction network rather than threshold-specific edges.

To examine potential scale effects of topological metrics, we applied min–max normalization to degree and intra-module density and recomputed the FerroScore in both the MEF and A549 datasets (Tables S1–S2, Fig. S6C). Normalization fully preserved the relative ranking of experimental conditions, indicating robustness to metric rescaling. Although statistical significance was attenuated in the MEF dataset, likely reflecting variance compression under limited sample size, correlations in A549 remained significant. Because normalization primarily rescales the FerroScore without altering its discriminative ordering and substantially compresses biologically meaningful structural variance in such small networks, we retained the original metric definitions for the main analyses and report the normalized results in the ***SI*** as a robustness assessment.

Finally, because FerroScore is initialized from DEGs defined relative to a control group, we further evaluated its sensitivity to the reference background by rerunning the complete pipeline under matched control leave-one-out and pooled control stress test settings (Table S3). A549 retained ferroptosis core modules and stable FerroScore values across the tested settings, whereas MEF showed stronger condition dependent sensitivity, particularly under pooled controls. These findings support FerroScore as a relative metric sensitive to the reference background and emphasize the importance of biologically matched controls for preserving interpretable network topology associated with ferroptosis.

### Dual roles of ferroptosis in pancreatic cancer patient survival

**Figure 3:**
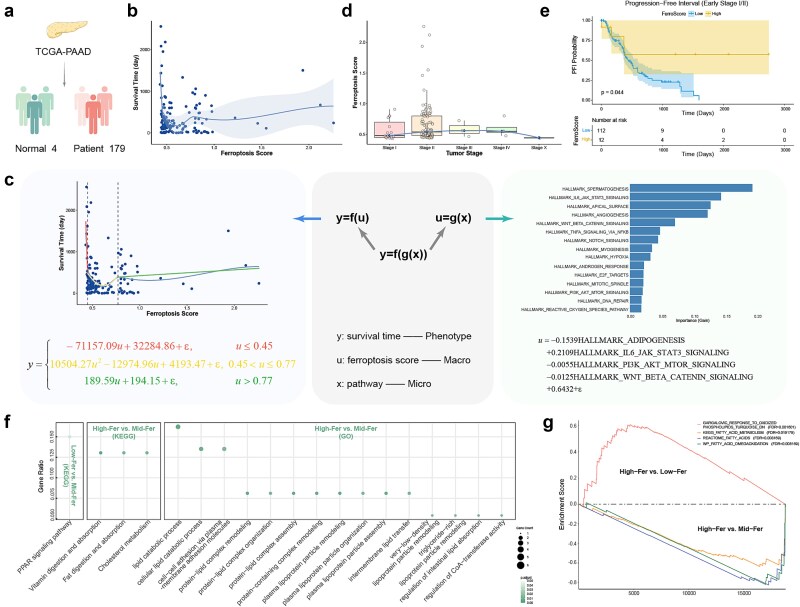
The dual role of ferroptosis in pancreatic cancer. (a) RNA-seq data from the TCGA-PAAD cohort, including 4 normal and 179 tumor samples. (b) Association between ferroptosis score and patient survival, with the 95% confidence interval shaded. (c) Analytical representation of the survival relationship as a composite function, linking pathway regulation (inner function) to ferroptosis activity and then to survival outcome (outer function). (d) Box plot of ferroptosis scores across tumor stages, with a fitted trendline. (e) Kaplan–Meier analysis of PFI for early-stage (stages I & II) patients. (f–g) Functional enrichment analyses comparing groups with different ferroptosis activity levels (Low-Fer, Mid-Fer, and High-Fer): GO and KEGG analyses (f), and GSEA enrichment analysis (g). Panel A was created using components from SciDraw.

We next applied FerroScore to bulk RNA-seq data to investigate the clinical relevance of ferroptosis at the individual level. Pancreatic adenocarcinoma (PAAD) was selected as the primary model due to its extensive metabolic reprogramming and hallmark resistance to conventional apoptosis-inducing therapies, features that collectively highlight the therapeutic potential of ferroptosis in this malignancy. Specifically, by RNA-seq data and clinical information from the TCGA PAAD cohort, including 179 tumors and 4 normal adjacent tissues (Fig. 3a), we calculated the ferroptosis score for each tumor sample, and its association with patient survival was analyzed. Locally weighted regression revealed a nonlinear U-shaped relationship between ferroptosis activity and survival (Fig. 3b). We observed that patients with low ferroptosis activity showed longer survival, possibly due to less aggressive, well-differentiated tumors. Similarly, high ferroptosis scores were associated with improved survival, likely reflecting extensive tumor cell death. In contrast, patients with intermediate ferroptosis levels had the poorest outcomes, suggesting that moderate activity may be insufficient to kill tumors while potentially activating pro-survival and metastatic pathways. This U-shaped pattern underscores the dual role of ferroptosis in the pancreatic cancer: moderate activity promotes malignancy, whereas high levels suppress tumors. These findings support therapeutic strategies aimed at inducing ferroptosis, as exemplified by the ferroptosis inducer Erastin, which has shown promise in preclinical and early clinical trials [27].

To further elucidate the underlying biological mechanisms, we parametrized the observed survival relationship as a composite function, $y=f\left(g(x)\right)$ (Fig. 3c). The outer function, $y=f(u)$, captures the triphasic impact of ferroptosis activity on survival: survival time decreased with increasing ferroptosis up to a point, exhibiting a U-shaped association across an intermediate range, and increased with further rises. The inner function, $u=g(x)$, characterizes how signaling pathways regulate ferroptosis activity. Using LightGBM and elastic net regression, we constructed an interpretable model for $g(x)$. This approach identified the spermatogenesis pathway as the most influential predictor, likely due to its high lipid metabolism, and delineated adipogenesis, PI3K-AKT-mTOR, and Wnt-β-catenin as suppressive pathways, while IL6-JAK-STAT3 promoted ferroptosis. These regulatory roles, as well as the dual role of ferroptosis in cancer, are consistent with the existing literatures [28–31]. This cross-scale framework integrally links pathway regulation to ferroptosis activity and clinical outcome, providing a foundation for developing precision ferroptosis therapies.

Tumor stage is a critical determinant of disease severity and patient prognosis. We investigated how ferroptosis activity varied with tumor stage and found that the median ferroptosis score followed an inverted U-shaped trajectory, peaking at stage III before declining in stage IV, suggesting an adaptive resistance mechanism in advanced disease (Fig. 3d). Stage-specific survival curves further revealed distinct nonlinear dynamics, with stage II presenting the broadest therapeutic window for ferroptosis-targeting interventions (Fig. S7A). To exclude confounding by tumor stage and evaluate the prognostic relevance of FerroScore, we further performed survival analysis restricted to early-stage (stages I–II) patients using progression-free interval (PFI) as the clinical endpoint. Patients with high FerroScore exhibited significantly prolonged PFI compared with the low-score group (*log-rank P = .044*; Fig. 3e), indicating a reduced risk of tumor progression or recurrence. This association supports the stage-independent prognostic value of FerroScore and suggests that elevated ferroptosis activity reflects a tumor-suppressive metabolic state. To delineate the evolving molecular mechanisms, we identified high-frequency consensus genes (Fig. S7B and C), enriched KEGG pathways (Fig. S7D), and GO terms (Fig. S7E) for each stage. This enabled the construction of a mechanistic map (Fig. S8), which revealed a dynamic evolution of ferroptosis: from simplicity in stage I, to high activity in stage II (driven by lipid peroxidation, GSH synthesis, and ROS), and stage III (sustained by iron metabolism with emerging resistance), culminating in suppression in stage IV alongside decreased free iron. This trajectory mirrored the pathological progression from metabolic reprogramming to uncontrolled proliferation. Our findings suggested that stages II–III represent the optimal therapeutic window for ferroptosis induction, whereas stage IV tumors shift reliance to alternative pathways such as endogenous hormone signaling. Therefore, for advanced-stage patients, treatment should prioritize targeting steroid synthesis to disrupt metabolic adaptability, followed by combination therapy with ferroptosis inducers for a synergistic effect.

We are also interested in exploring the reasons for the differences in ferroptosis activity. Based on the previously defined score cutoffs, we stratified patients into Low-, Mid-, and High-Fer groups. Functional enrichment analyses (GO, KEGG, GSEA) revealed a strong association between the ferroptosis activity gradient and lipid metabolic reprogramming (Fig. 3f and g). A key finding was the biphasic regulation of lipid utilization in the High-Fer group: enhanced upstream lipid catabolism (GO results), potentially supplying polyunsaturated fatty acids (PUFAs) as ferroptosis substrates, coupled with suppressed downstream fatty acid oxidation (GSEA results), which may preserve these PUFAs from being catabolized. This reprogramming maintained pools of peroxidation-ready PUFAs, directing lipids toward membrane restructuring and peroxidation rather than energy supply. Conversely, the Low-Fer group appeared to suppress ferroptosis by activating PPAR signaling and enhancing antioxidant defense. These findings illuminate lipid metabolism as a promising target for inducing ferroptosis. Finally, decision curve analysis demonstrated that our ferroptosis index provided a higher net benefit than traditional tumor staging across a wide threshold probability range ($\ge 0.2$), supporting its potential clinical utility (Fig. S7F).

### Tumor-associated macrophages and neural-modulatory fibroblasts in the tumor microenvironment exhibit high ferroptosis activity

**Figure 4:**
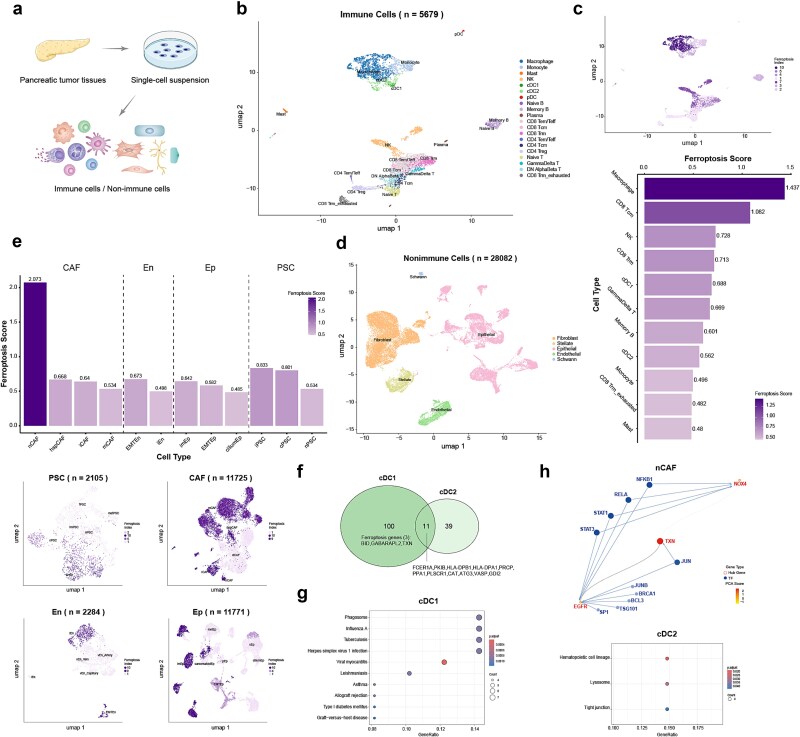
Identification and the molecular mechanisms of stromal cells with high ferroptosis activity in the TME. (a) Analytical workflow for scRNA-seq data from pancreatic tumors. (b) UMAP visualization of immune cell subtypes. (c) Ferroptosis activity in immune cells: UMAP plot showing the ferroptosis index (top) and bar plot showing ferroptosis scores per subtype (bottom). Higher index values represent higher activity. (d) UMAP visualization of non-immune cell subtypes. (e) Ferroptosis scores (top) and ferroptosis index (bottom) for PSC, CAF, En, and Ep subclusters. (f) Gene sets for cDC1 and cDC2 comparison: subtype-specific, shared, and known ferroptosis-related genes. (g) KEGG pathway enrichment for cDC1- and cDC2-specific genes. (h) TF-target gene regulatory network in nCAFs. Red nodes represent key driver genes; blue nodes denote TFs. Node size reflects the strength of regulatory interactions. Panel A was created using components from SciDraw.

To characterize ferroptosis at the cellular level in the tumor microenvironment (TME), we analyzed scRNA-seq data. Specifically, we profiled immune and non-immune cells from 3 and 17 pancreatic tumor tissues, respectively [32] (Fig. 4a). Cell annotation identified 20 immune cell subtypes (Figs 4b, S9A) (see ***SI*** for details). Within the myeloid lineage, we identified a dominant population of Tumor-Associated Macrophages (TAMs), defined by the high expression of canonical markers (e.g. CD163, C1QC). These TAMs, along with CD8 Tcm and NK cells, exhibited high ferroptosis activity, suggesting a functional role for ferroptosis in Macrophage polarization, T cell memory maintenance, and NK cell regulation (Fig. 4c). We observed pronounced differences in ferroptosis between CD8 Trm and CD8 Trm_exhausted cells, as well as between cDC1 and cDC2 subsets, indicating heterogeneous regulation across different functional states. For non-immune cells, initial classification into five broad types (Figs 4d, S9B) revealed limited discriminative capacity of ferroptosis index (Fig. S10A). To resolve this, we subclustered the Epithelial, Endothelial, Fibroblast, and Stellate populations based on functional characteristics and marker specificity (Figs S9C–F, S10B–E) (see ***SI*** for details) and re-assessed ferroptosis activity at this refined resolution (Fig. 4e). Subclustering of the fibroblast population revealed a distinct subset characterized by the specific enrichment of “peripheral nervous system development” and “axonogenesis” pathways, which we termed Neural-modulatory CAFs (nCAFs). nCAFs displayed heightened ferroptosis susceptibility and were distinct from other CAF subtypes. Alongside the previously reported FerroCAFs [33], nCAFs represent a novel ferroptosis-prone CAF. Elevated ferroptosis activity was also detected in immune-modulated subtypes (e.g. iCAF, iEn, imEp, iPSC), EMT-related subtypes (EMTEn, EMTEp) and cPSC cells. In line with prior evidence that mesenchymal-like cancer cells were more prone to ferroptosis than epithelial-like cells [34], we showed that EMTEn cells had higher ferroptosis scores than EMTEp cells, hinting a role of ferroptosis in epithelial–mesenchymal transitions.

We next investigated the mechanisms underlying differential ferroptosis susceptibility across cell states. Taking cDC1 and cDC2 cells as an example, we identified subtype-specific, shared, and known ferroptosis-related DEGs (Fig. 4f) and performed KEGG enrichment analysis on the subtype-specific gene sets (Fig. 4g). Although previous studies reported that DCs can undergo ferroptosis in the TME [35], the heterogeneity in susceptibility among different DC subsets and the underlying mechanisms remain unclear. Our analysis revealed that cDC1s upregulated pro-ferroptotic genes (e.g. BID, TXN) and were enriched in phagosome and viral response pathways, suggesting that a high antigen-presenting state, along with associated ROS accumulation, underlay their ferroptosis susceptibility. This finding resonated with prior work on the “Zeb1–microRNA-96/182–Cybb axis” which reported cDC1 characteristics of ROS generation in phagosomes [36]. In contrast, cDC2s exhibited enhanced lysosomal and cell junction pathways, supporting iron homeostasis and ferroptosis resistance. In addition, a parallel analysis of CD8 Trm and exhausted T cells revealed that exhausted cells upregulated a KLF2-mediated antioxidant program, reducing ferroptosis susceptibility to promote survival (Fig. S11A–B). These findings demonstrated how distinct immune cell states differentially adapted to the TME through ferroptosis regulation.

In non-immune cells, nCAFs showed the highest ferroptosis susceptibility. To elucidate the underlying regulatory mechanisms, we constructed a transcription factor (TF)–target gene regulatory network (Figs 4h, S11C), which identified TXN, EGFR, and NOX4 as core drivers modulated by TFs including NFKB1, STAT1, RELA, STAT3, and JUN. Mechanistically, ferroptosis in nCAFs resulted from an imbalance between pro-death signals (e.g. NOX4–ROS) and antioxidant compensatory mechanisms (e.g. TXN), orchestrated by a transcriptional network centered on NF-κB and STAT families. Key drivers included NOX4, whose expression was promoted by NFKB1/RELA/STAT1/STAT3 under inflammatory stress, directly increasing ROS and lipid peroxidation. Stress-responsive TFs also upregulated EGFR, which exacerbated redox imbalance by depleting antioxidants. In opposition, the JUN-regulated TXN system served as a pivotal compensatory antioxidant mechanism, and ferroptosis ensued when this TXN-mediated defense failed. Targeting this balance, such as by inhibiting TXN or enhancing NOX4/EGFR signaling, presents a potential strategy for nCAF-targeted therapy. We also observed divergent ferroptosis susceptibility between EMTEn and EMTEp cells (Fig. S11D–E), which reflected their distinct microenvironmental niches. EMTEn cells, enriched in hypoxia and vascular contraction pathways, likely resided in oxidative stress-prone invasive fronts. By contrast, EMTEp cells, active in extracellular matrix and adhesion pathways, occupied the structured tumor core and maintained robust antioxidant defenses. These results collectively highlight the heterogeneity of ferroptosis susceptibility among tumor stromal cells, providing new avenues for stromal-specific therapy in pancreatic cancer.

### Ferroptosis drives an immunosuppressive microenvironment through reprogramming and intercellular communication

Our earlier work identified high ferroptosis activity in cell subsets such as Macrophages, CD8 Tcm cells, and nCAFs, indicating that ferroptosis may be deeply involved in core processes of tumor immune regulation. Guided by this finding, we pursued a deeper immune investigation into how ferroptosis shapes immune cell functionality and how high ferroptosis cells remodel the TME via intercellular communication.

We first investigated how ferroptosis sensitivity alters immune function in Macrophages and CD8 Tcm cells. GSEA of ferroptosis- and immune-related pathways (Fig. S12A and D), combined with TF–target network analysis (Fig. S12B–C, E), revealed distinct immunoregulatory mechanisms. In Macrophages, ferroptosis susceptibility was driven by altered lipid metabolism and compromised antioxidant defense. GSEA indicated activated fatty acid transport and sphingolipid degradation alongside suppressed membrane-stabilizing phospholipid metabolism, promoting the accumulation of lipid peroxidation substrates. The regulatory network showed that insufficient GPX4 expression—regulated by CREM and TFAP2A—led to defective lipid peroxide clearance. Moreover, ferroptosis-prone Macrophages underwent extensive immunoregulatory reprogramming. Pro-inflammatory TFs (RELA, NFKB1, STAT3) upregulated immunosuppressive factors like IL1RN, while SPI1-regulated CTSS and VDR/SP2/SP3-co-regulated CD14 collectively reinforced an immunosuppressive phenotype. This reprogramming suppressed adaptive immune pathways and antigen presentation, polarizing Macrophages toward a tolerant state that fosters immune escape—consistent with reported M2-like Macrophage traits and their heightened ferroptosis vulnerability relative to M1 states [37–39]. Our results also confirmed the characteristically high ferroptosis activity of CD8 T cells [40, 41], while specifically revealing that in the CD8 Tcm subset, this activity correlated with disrupted membrane lipid homeostasis and impaired effector function, modulated by a BRD7–BRD2 epigenetic module that may promote immune suppression through metabolic-epigenetic remodeling. Targeting these cell-type-specific regulatory axes could help revert immunosuppression in both Macrophages and CD8 Tcm cells, potentially synergizing with existing immune checkpoint inhibitors.

**Figure 5:**
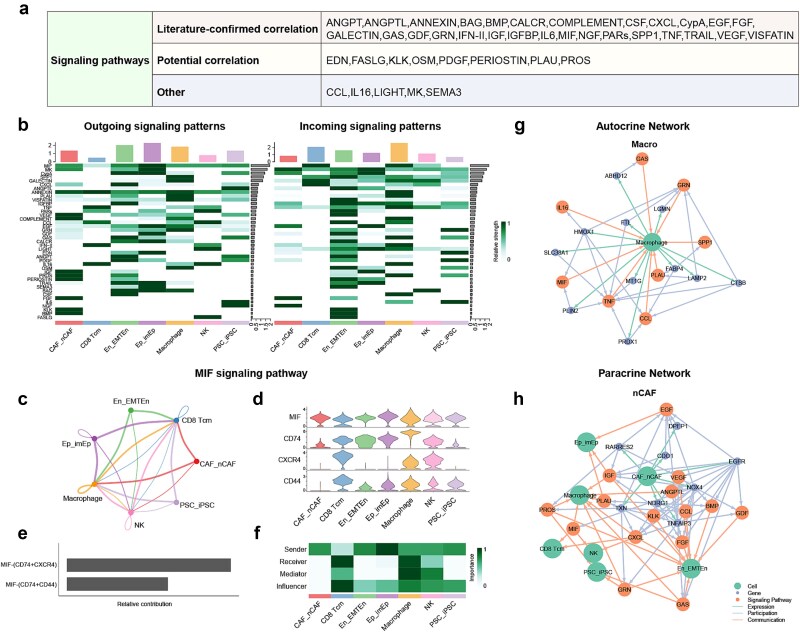
Role and regulatory mechanisms of ferroptosis in remodeling the tumor immune microenvironment. (a) Signaling pathways among high-ferroptosis cell subtypes, categorized by ferroptosis association. (b) Contribution of signaling pathways to intercellular communication. (c–f) MIF signaling among high-ferroptosis cells: (c) communication network (edge thickness reflects communication strength). (d) Gene expression patterns. (e) Ligand–receptor pair contributions. (f) Functional roles of subtypes (sender, receiver, mediator, influencer). (g–h) Ferroptosis “cell-gene-signal” regulatory network showing autocrine (g, Macrophage) and paracrine (h, nCAF) loops.

Beyond cell-intrinsic changes, intercellular communication critically shapes the TME. To systematically delineate interactions among cells with high ferroptosis activity and their microenvironmental impact, we analyzed seven key subtypes—Macrophages, CD8 Tcm, NK cells, nCAF, iPSC, EMTEn, and imEp—using CellChat [42, 43]. We identified active signaling pathways and categorized them by their relevance to ferroptosis (Fig. 5a, Table S4). Signal contribution analysis (Fig. 5b) identified MIF, MK, CyPA, SPP1, GALECTIN, and CXCL as key pathways in ferroptosis-driven TME reprogramming. imEp cells emerged as the dominant signal senders, particularly through MIF and MK pathways, while Macrophages acted as primary receivers and responders. We focused on five key pathways—MIF, GALECTIN, SPP1, PLAU, and MK—for in-depth analysis, examining their network structures, gene expression, ligand–receptor contributions, and key communicating cells (Figs 5c–f, S13A–D). The MIF pathway, primarily sent by imEp and received by CD8 Tcm via CD74–CXCR4, was critically modulated by Macrophages, suggesting a mechanism for epithelial-driven T cell suppression under Macrophage control. Similarly, the GALECTIN pathway, dominated by Macrophages through LGALS9–P4HB, strongly inhibited CD8 T cells and promoted immune escape. Beyond immunosuppressive pathways, we uncovered a Macrophage-driven pro-invasive pathway mediated by SPP1. Aligning with a recent study highlighting its role in mesenchymal transition [44], Macrophage-derived SPP1 activated EMT-like cells via CD44, demonstrating how immune cells potentiate the core SPP1–CD61–BMP2 axis to promote metastatic dissemination. Besides, PLAU and MK pathways displayed autocrine characteristics. Macrophages utilized PLAU for self-regulation, while imEp cells employed an MDK–NCL loop to sustain survival. In summary, these results reveal that ferroptotic cells actively remodel the TME toward immunosuppression and pro-metastatic traits via specific cell–cell communication events. This process is amplified by autocrine signals that form self-reinforcing circuits, further accelerating malignant progression.

Finally, to delineate how ferroptosis signals are transmitted across the TME, we constructed a “Cell–Gene–Signal” multilayer network (Figs 5g–h, S13E–F) by integrating cell-specific ferroptosis genes, intercellular communication data, and gene–pathway associations. This framework models high-ferroptosis cells, their DEGs, and active pathways as interconnected nodes, with edges based on expression significance, pathway membership, and communication probability. Analysis of complete circuits revealed both autocrine and paracrine ferroptosis loops, mapping signal routes across cellular and molecular tiers. The results showed that Macrophage, nCAF, iPSC, EMTEn, and imEp cells formed autocrine regulatory circuits, with Macrophage displaying the most complex structure. All high-ferroptosis cell types except CD8 Tcm participated in paracrine signaling, with nCAF, Macrophage, iPSC, and imEp cells forming particularly robust connections. These transmission paths elucidate fundamental ferroptosis mechanisms and highlight their translational relevance for cancer immunotherapy. For example, the “Macrophage–HMOX1–TNF–Macrophage” circuit represents a core regulatory axis in which ferroptosis and inflammation mutually reinforce each other. The multilayer network provides systematic insights and potential targets for developing ferroptosis-modulating therapies tailored to the TME.

### Ferroptosis accelerates the pathogenesis of Alzheimer’s disease

Ferroptosis is driven by iron accumulation, lipid peroxidation, and GSH depletion, representing metabolic features that are also central to neurodegeneration. Owing to its high oxygen consumption and enrichment in PUFAs, the brain is intrinsically vulnerable to iron-dependent oxidative damage, supporting ferroptosis as a conserved pathological mechanism beyond oncology [45–47]. Taking AD as an example, we identified an “inverted–checkmark” relationship between ferroptosis score and age at death, linking elevated ferroptosis to accelerated mortality (Fig. S14A). Correlation analyses with AD pathological markers revealed positive associations between ferroptosis score and both APOE genotype and Thal phase, implicating the APOE4 risk variant and Aβ deposition in promoting ferroptosis (Fig. S14B–D). Conversely, a “reverse inverted-checkmark” pattern with Braak staging indicated that tau pathology drives ferroptosis primarily during early to mid-disease stages. Furthermore, a stage-progressive map of ferroptosis mechanisms across Braak stages (Fig. S14E) nominated stage V.5 as a potential intervention window. These findings collectively support ferroptosis inhibition as a promising therapeutic strategy for AD.

## Conclusion

Ferroptosis, an iron-dependent form of non-apoptotic cell death, represents a promising therapeutic avenue for treatment-resistant cancers. However, existing detection techniques are limited by their throughput, cost, and resolution. Here, we developed FerroScore, a computational tool that quantified ferroptosis activity from transcriptomic data at both bulk and single-cell resolution. By integrating core molecular features with network topology, FerroScore provided quantitative activity assessments alongside mechanistic interpretations, enabling a deeper understanding of ferroptosis in cancer biology.

Through benchmarking in cell lines, pancreatic cancer (bulk and single-cell) and Alzheimer’s transcriptomes, FerroScore proved robust and generalizable. Subsequent analyses highlighted its utility in revealing ferroptosis as a therapeutically inducible process in cancer, a suppressible pathway in neurodegeneration, and a mediator of immunosuppressive TME remodeling.

To further assess its scalability, we extended FerroScore to independent datasets including Liver Hepatocellular Carcinoma (LIHC), Kidney Renal Clear Cell Carcinoma (KIRC), cardiomyopathy cohorts (ischemic cardiomyopathy, ICM, and dilated cardiomyopathy, DCM), and Nonalcoholic Fatty Liver Disease (NAFLD) (Fig. S15). Across these heterogeneous pathological contexts, FerroScore consistently captured disease-relevant ferroptotic alterations and exhibited prognostic or pathological stratification capacity, underscoring its broad cross-disease applicability.

It should be noted that FerroScore is primarily built upon three core metabolic mechanisms: iron, GSH, and lipid metabolism. However, other pathways (such as the ESCRT-III membrane repair system [48] and the DHODH pathway [49]) also regulate ferroptosis. Incorporating these pathways in future developments may substantially expand the tool’s representational scope and predictive value. Moreover, while our intercellular communication analysis inferred spatially dependent paracrine signaling (e.g. between nCAFs and tumor cells), the precise *in situ* architecture of these subpopulations remains to be directly visualized. Integrating spatial transcriptomics represents a critical next step, and our ongoing work is dedicated to constructing a spatial heterogeneity atlas to resolve the microenvironmental organization of ferroptosis-active niches. Additionally, to evaluate the contributions of individual functional modules, one may employ a multi-level analytical strategy of global module architecture and node-level properties, which integrates network topology metrics including modularity, intra-module density, degree, betweenness centrality, and clustering coefficient, as done in this paper. Future refinements of the scoring framework here are expected to enhance its predictive performance and biological interpretability. Finally, while FerroScore provides multi-scale insights into ferroptosis regulation in PAAD and AD, our extended analyses underscore that its biological interpretation is inherently context-dependent. This is manifested through the divergent stage-associated patterns observed in LIHC and KIRC, indicating that ferroptosis dynamics are uniquely shaped by tumor-specific metabolic landscapes and pathological progressions. We further identify a potential limitation in fibrotic or advanced remodeling states (such as ICM), where the substantial replacement of functional parenchyma by extracellular matrix may attenuate ferroptotic transcriptomic signals, resulting in a signal dilution effect in bulk RNA-seq data. Accounting for such tissue composition and disease stage is imperative for precise score interpretation. Nonetheless, by establishing a scalable and mechanistically interpretable framework capable of resolving both shared and disease-specific regulatory programs, FerroScore advances the mechanistic understanding of ferroptosis-associated pathologies and provides a robust foundation for the translational development of ferroptosis-targeted precision therapies.

**Key Points**

- FerroScore, a novel statistical, is proposed to quantify the ferroptosis activity from transcriptomic data at both bulk-tissue and single-cell resolution, overcoming the limitations of conventional experimental assays.
- FerroScore integrates core pathway activities (iron, glutathione, and lipid metabolism) with network topologies to generate an interpretable ferroptosis score.
- Applied to pancreatic-cancer data, FerroScore uncovers a U-shaped ferroptosis–survival relationship, cell-type-specific activity (Macrophages, CD8 Tcm, nCAFs), and TME remodeling driven by immunosuppressive and pro-metastatic MIF, GALECTIN, and SPP1 signaling.

## Supplementary Material

Revised_SI_No_BIB-25-2407_R2_bbag368

## Data Availability

The mouse MEF cell line RNA-seq data used in this study are available from the Gene Expression Omnibus (GEO; https://www.ncbi.nlm.nih.gov/geo/) under accession number GSE237928. The human A549 cell line RNA-seq data can be obtained from the Sequence Read Archive (SRA; https://www.ncbi.nlm.nih.gov/sra) under accession number PRJNA979805. Bulk RNA-seq data and corresponding clinical information for PAAD, as well as for LIHC and KIRC, are publicly accessible via The Cancer Genome Atlas (TCGA; https://portal.gdc.cancer.gov/). scRNA-seq data of immune and non-immune cells from pancreatic tumor tissues are downloaded from the GEO database under accession numbers GSE235449 and GSE194247, respectively. RNA-seq data from cardiomyopathy cohorts, including ICM and DCM, are obtained from GEO under accession number GSE116250. Transcriptomic data for NAFLD are retrieved from GEO under accession number GSE135251.RNA-seq and clinical data for Alzheimer’s disease are sourced from The Mayo RNAseq Study (MayoRNAseq) project within the AD Knowledge Portal (https://adknowledgeportal.org). The AD Knowledge Portal is a data-sharing platform supported by the Accelerating Medicines Partnership–Alzheimer’s Disease (AMP-AD) and the National Institute on Aging (NIA); data access requires application and approval.

## Code availability

FerroScore is publicly available as an R package. The benchmark, as well as source codes have been deposited at the GitHub repository (https://github.com/tjq3/FerroScore).
