# Supplementary material for: FerroScore: a statistical approach for quantifying tumor-related ferroptosis based on omics data
Source: Brief Bioinform. 2026 Jul 6;27(4):bbag368. doi: 10.1093/bib/bbag368 (PMC13336637; doi:10.1093/bib/bbag368)
Supplement: Revised_SI_No_BIB-25-2407_R2_bbag368 [file revised_si_no_bib-25-2407_r2_bbag368.docx]

**Supplementary Information for**

**FerroScore: A statistical approach for quantifying tumor-related ferroptosis based on omics data**

Jiaqi Teng, Qi Gong, Zhaohang Cai, Tianshou Zhou*

School of Mathematics, Sun Yat-sen University, Guangzhou 510275, Guangdong Province, China

*The corresponding author: [mcszhtsh@mail.sysu.edu.cn](mailto:mcszhtsh@mail.sysu.edu.cn)

**This PDF file includes:**

Supplementary text

Figures S1 to S15

Table S1 to S4

**Supplementary Information Text**

**Materials and Methods**

**Data Preprocessing**

**Bulk RNA-sequencing Data.** Raw RNA-seq data used in our study were downloaded from the TCGA-PAAD cohort, which comprise 183 samples (4 adjacent normal tissues and 179 pancreatic tumor tissues). First, the *.json* metadata files from the TCGA database were parsed to establish sample‐to‐file mappings. Then, all raw count files in *.tsv* format were read to extract unnormalized raw counts. Third, gene identifiers were uniformly converted to Gene Symbols, and for duplicate genes, the maximum expression value was retained. Finally, only protein‐coding genes were preserved, thus obtaining an expression matrix containing 19937 genes, which served as the input for FerroScore.

**Single-cell RNA-sequencing Data.** The scRNA-seq data of pancreatic cancer immune cells were obtained from the GEO dataset GSE235449. Transcript counts were first aggregated to the gene level to create a Seurat object. A standard preprocessing workflow was then performed, including quality control filtering, data normalization, highly variable feature selection, data scaling, PCA-based linear dimensionality reduction, cell clustering, and UMAP-based nonlinear dimensionality reduction. Quality control criteria were set as follows: *UMI* > 2000, *gene count* between 500-7000, *mitochondrial gene percentage* < 10%, and doublets were removed using scDblFinder. Normalization was performed using the LogNormalize method with a scale factor of 10000. The top 3000 highly variable genes were selected based on the vst method, followed by data scaling. PCA linear dimensionality reduction, cell clustering, and UMAP nonlinear dimensionality reduction were conducted using the first 21 principal components determined by the elbow plot. This preprocessing yielded a high-quality immune cell dataset containing 5679 cells and 30397 genes. For the non-immune cell dataset (GSE194247), the same preprocessing workflow was applied, except that the number of principal components used for dimensionality reduction and clustering was adjusted to 20 based on its elbow plot, obtaining a final non-immune cell dataset of 28082 cells and 30397 genes.

**Construction of the FerroScore and Ferroptosis Index**

**Step 1 Differential Expression Analysis.** Raw RNA‑seq or scRNA‑seq data were preprocessed through identifier conversion, quality control (retaining genes with *CPM* > 100 in at least one sample), and normalization (using the TMM method to mitigate inter-sample biases). Differential expression analysis was then performed using edgeR, i.e., by comparing target samples (Treated) against biologically relevant reference groups (Control). The definitions of the two groups depend on study design. Specifically, for RNA-seq datasets, adjacent normal tissues or healthy controls served as the reference group, while disease samples constituted the target group. For scRNA-seq datasets, cells of interest were designated as the target group, whereas all remaining cell populations were used as the reference group, representing the transcriptomic baseline of the cellular microenvironment. Genes with *p‑value* < 0.05 and *|logFC|* > 1 were defined as DEGs. A ferroptosis‑related gene set was compiled from the FerrDb database, and Venn analysis was employed to determine its overlap between these genes and the DEGs. GO and KEGG functional enrichment analysis was performed on the resulting intersection to elucidate the biological functions and pathway features associated with ferroptosis‑related differential expression. In cases where no significant overlap was detected, the same enrichment analysis was performed on the full set of DEGs to explore potential regulatory mechanisms, thereby completing the initial exploration of the data.

**Step 2 Protein–Protein Interaction Network Construction.** Using the STRING database, DEGs identified in Step 1 were mapped to their corresponding proteins, and PPI data were retrieved. Only those interacting pairs that involved these proteins were retained, with an interaction *confidence score threshold* set at $\geq400$. The resulting proteins, mapped from the DEGs, were treated as nodes in the network, while the filtered high-confidence interaction pairs formed the edges. This process yielded an undirected PPI network convenient to subsequent analysis.

**Step 3 Module Identification and Annotation.** The Louvain algorithm [1,2] is a community detection method based on modularity optimization. It identifies an optimal partition of the network through iterative modularity optimization and community merging. A modularity, $Q$, is defined as

$$Q=\frac{1}{2m}\sum_{i,j} \left(A_{ij}-\frac{k_{i}k_{j}}{2m}\right),$$

and the modularity gain, $\Delta Q$, is given by

$$\Delta Q=\left[\frac{\sum_{\text{in}}+2k_{i,\text{in}}}{2m}-\left(\frac{\sum_{\text{tot}}+k_{i}}{2m}\right)^{2}\right]-\left[\frac{\sum_{\text{in}}}{2m}-\left(\frac{\sum_{\text{tot}}}{2m}\right)^{2}-\left(\frac{k_{i}}{2m}\right)^{2}\right],$$

where  denotes the sum of edge weights within module $c$;  represents the sum of edge weights connected to nodes in module $c$ (including edges linking to other modules); $k_{i}$ is the sum of edge weights connected to node $i$; $k_{i,\text{in}}$ refers to the sum of edge weights between node $i$ and nodes in module $c$; and $m$ stands for the total weight of all edges in the network.

However, the Louvain algorithm may produce disconnected communities. The Leiden algorithm overcomes this limitation through an extended modularity formulation that includes a resolution parameter $\gamma$ to adjust the density of intra‑ and inter‑community links

$$Q=\frac{1}{2m}\sum_{i,j} \left(A_{ij}-\gamma\frac{k_{i}k_{j}}{2m}\right).$$

A resolution parameter $\gamma>1$ promotes the formation of more numerous, smaller, and more tightly connected communities, whereas $0<\gamma<1$ results in fewer, larger, and more loosely linked communities. It offers superior performance over Louvain in terms of connectivity, partition quality, and convergence. In this study, we partitioned the PPI network from Step 2 using the Leiden algorithm with the $\gamma$ value set to 1.

Following module partitioning of the PPI network, each module was subjected to GO and KEGG functional enrichment analysis to identify significantly enriched biological processes and pathways. The detection logic was implemented as follows: enrichment was first performed for GO_BP and KEGG terms; if no significant results were obtained, the analysis was then extended to GO_ALL and KEGG. Based on the enrichment results, modules were automatically named using a predefined keyword set comprising lipid-, GSH-, and iron metabolism-related terms. The modules matching these keywords received a categorical designation (e.g., "Lipid/GSH/Iron metabolism"); otherwise, the most significant functional term identified in the enrichment analysis was assigned as the module name.

**Step 4 Topological Feature Extraction.** Each of the aforementioned modules was further aggregated into super-nodes to construct a condensed network representation. From this network, nodes associated with iron metabolism, lipid metabolism, and GSH metabolism were extracted to assemble the final ferroptosis network. For each super-node in the ferroptosis network, its features were quantified at two levels: the module structure within the modularized PPI network, and the node-level attributes in the new network.

For the structure of module $c$ in the modularized PPI network, we used

1. Modularity, which quantifies the quality of network modularization, defined as follows

$$Q(c)=\frac{1}{2m}\sum_{i,j\in c} \left(A_{ij}-\frac{k_{i}k_{j}}{2m}\right)=\frac{\sum_{\text{in}}}{2m}-\left(\frac{\sum_{\text{tot}}}{2m}\right)^{2},$$

where $A_{ij}$ denotes the edge weight between nodes $i$ and$j$ (equal to 1 in unweighted networks); $k_{i}=\sum_{j} A_{ij}$ represents the sum of edge weights connected to node $i$; $m=\frac{1}{2}\sum_{ij} A_{ij}$ is the total weight of all edges in the network;  refers to the sum of edge weights within module $c$;  indicates the total weight of edges connected to nodes in module $c$. Although the theoretical range of $Q\left(c\right)$ is [−0.5, 1), its empirical values in the present study remained close to zero due to the highly compressed super-node topology containing only three module-level nodes.

1. Intra-module Density, which measures the compactness of connections within module $c$, defined as:

$$D(c)=\frac{2\times E_{c}}{N_{c}\left(N_{c}-1\right)},$$

where $E_{c}$ denotes the number of edges within module $c$; $N_{c}$ represents the number of nodes within module $c$. Due to the retention of symmetric PPI edges, $D\left(c\right)$ empirically ranged within (0, 2] in this study.

For the topological properties of node $i$ in the new network, we adopted

1. Degree, reflecting node connectivity, defined as

$$k_{i}=\sum_{j=1}^{N} A_{ij},$$

where $A_{ij}$ is defined as above, representing the connection relationship between nodes $i$ and $j$. Self-loops were retained to reflect aggregation of multiple underlying genes into each super-node, resulting in discrete degree values $k_{i}\in\left\{2,3,4\right\}$.

1. Betweenness centrality, quantifying the extent to which a node mediates information flow between modules, defined as

$$BC(i)=\sum_{j\neq i\neq k} \frac{\sigma_{jk}(i)}{\sigma_{jk}},$$

where $\sigma_{jk}$ represents the total number of shortest paths between nodes $j$ and $k$;$\sigma_{jk}(i)$ is the number of those shortest paths that pass through node $i$. In this three-module network, $BC_{i}$ was assumed to take binary values {0,1}, indicating whether a metabolic module serves as an inter-module bridge.

1. Clustering coefficient, measuring the tendency of a node’s neighbors to form closed triads, defined as

$$C(i)=\frac{2E_{i}}{k_{i}(k_{i}-1)},$$

where $E_{i}$ denotes the number of edges actually present among the neighbors of the node; $k_{i}\left(k_{i}-1\right)/2$ represents the maximum possible number of edges among those neighbors; and $k_{i}$ is the degree of node $i$. Here, $C\left(i\right)$ also took values in {0,1}, reflecting the presence or absence of tri-module closure among iron, lipid, and GSH pathways.

Given that these five metrics lie within comparable ranges and describe complementary dimensions of the same metabolic super-node, encompassing internal module organization ($Q\left(c\right),D\left(c\right)$) and external topological roles ($k_{i},BC\left(i\right),C\left(i\right)$), super-node $i$ was consequently evaluated by directly averaging them. This approach captures a composite structural phenotype that integrates intra-pathway cohesion with inter-pathway coordination:

$$\text{super node}_{i}\text{=}\frac{Q(c)+D(c)+k_{i}+BC(i)+C(i)}{5}.$$

**Step 5 Ferroptosis Score Calculation.** The formulation of the ferroptosis score was based on the molecular interplay among iron, lipid, and GSH metabolism, representing a mechanism-driven model guided by established biological priors. It explicitly captured the synergistic and antagonistic relationships among the three core axes of ferroptosis. Biologically, ferroptosis is characterized by iron-dependent lipid peroxidation, in which iron acts as the catalytic driver for ROS generation and polyunsaturated lipids serve as peroxidizable substrates. Together, they function as cooperative pro-ferroptotic drivers. Conversely, the GSH–GPX4 axis constitutes the primary antioxidant defense, functioning as an anti-ferroptotic brake that constrains lipid peroxide accumulation. Accordingly, the ferroptosis score was defined using a multiplicative–divisive structure:

$$\text{Ferroptosis score=}\frac{\text{Iron}\cdot\text{Lipid}}{\text{GSH}},$$

where *Iron*, *Lipid*, and *GSH* denote the topological activity scores of the corresponding metabolic super-nodes derived from Step 4. Notably, these topological scores quantify pathway-level functional integrity rather than transcriptional directionality (e.g., log-fold change). Within this framework, functional suppression of the protective GSH pathway is encoded as reduced intra-module cohesion and weakened inter-module connectivity (i.e., topological disintegration), leading to a decreased GSH denominator and consequently an increased FerroScore. This formulation enabled the FerroScore to capture pathway-level loss-of-function phenotypes through coordinated structural degradation, rather than relying on the expression polarity of individual genes.

The multiplicative term ($\text{Iron}\cdot\text{Lipid}$) reflected the requirement for synergistic activation, ensuring that a deficiency in either component suppressed ferroptotic propensity, consistent with the law of mass action. This formulation avoided biologically implausible outcomes permitted by additive models (e.g., elevated iron alone yielding high scores in the absence of oxidizable lipids). Weighted linear models were deliberately avoided to preserve mechanistic interpretability and prevent dataset-specific overfitting. This parameter-free design supported robust transferability across disease contexts by relying on conserved ferroptotic metabolic logic rather than training-dependent weights.

In addition, to ensure robustness to incomplete data, a neutral value of 1 (identity element) was assigned to any missing metabolic category, preserving contributions from remaining active modules and enabling reliable score calculation across heterogeneous network compositions.

**Step 6 Ferroptosis Index Grading.** A sufficient number of ferroptosis scores are collected as training samples. Let $S=\{s_{1},s_{2},\cdots,s_{n}\}$ represent the set of ferroptosis scores in the training sample, where . The valid score set is then denoted as $S_{\text{valid}}=\{s\in S|s\neq\text{NA}\}$.

The ferroptosis index is categorized using a quantile-based approach. First, the decile cutoff points of the sample are calculated according to

$$Q=\left(q_{0},q_{0.1},\cdots,q_{1}\right)=\mathrm{quantile}\left(S_{\text{valid}},p\right),$$

where $p\in\{0,0.1,\cdots,1\}$ and $q_{p}$ satisfies $P\left(S_{\text{valid}}\leq q_{p}\right)\geq p$. Then, a discretization function  was defined to map the ferroptosis score to a ferroptosis index (on a 1–10 scale), i.e.,

$$f(s)=\left\{\begin{matrix} 1\text{ if }s\text{ = NA} \\ \max\{k\in\{1,2,\cdots,10\}|s\geq q_{\frac{k-1}{10}}\}\text{ otherwise} \end{matrix}\right..$$

Thus, for any test sample $x$, its ferroptosis index is given by

$$\text{Ferroptosis index}\left(x\right)\text{ =}\left\{\begin{matrix} 10\text{ if }x\geq q_{0.9}, \\ 9\text{ if }q_{0.8}\leq x<q_{0.9}, \\ \vdots\\ \text{2 if }q_{0.1}\leq x<q_{0.2}, \\ 1\text{ if }x<q_{0.1}\text{ or }x=\text{NA.} \end{matrix}\right.$$

This quantile-based ferroptosis index possesses two key properties: uniformity in grade distribution, that is, probability $P\left(f(s)=k\right)\approx0.1$ for$\forall k\in\{1,2,\cdots,10\}$, and monotonicity, i.e., $f(x_{1})\geq f(x_{2})$ for $\forall x_{1}\geq x_{2}$. The index satisfies the boundary conditions$\lim_{x\to-\infty} f(x)=1$ and $f(\max(S_{\text{valid}}))=10$, ensuring adaptability to non-uniform data distributions.

For any individual or cell as a test sample, the above process returns a ferroptosis score and index. FerroScore will skip the calculation and return an NA value under the following cases: 1. Fewer than 5 DEGs are identified; 2. A functional module contains zero genes; 3. None of the three core ferroptosis metabolic modules (iron, lipid, and GSH metabolism) are present.

**Computational Details**

**R Package Versions.** All computational analyses were performed within the R statistical computing environment (v4.3.2). The specific versions of the key R packages required for the FerroScore algorithm and associated analyses are listed below: edgeR (v4.0.16), igraph (v2.2.1), STRINGdb (v2.14.3), leidenbase (v0.1.36), clusterProfiler (v4.10.1), org.Hs.eg.db (v3.18.0), org.Mm.eg.db (v3.18.0), openxlsx (v4.2.8.1), Seurat (v5.4.0), scDblFinder (v1.16.0), tidyverse (v2.0.0), GSVA (v1.50.5), survival (v3.8.3), glmnet (v4.1.10), mgcv (v1.9.0), lightgbm (v4.6.0), minpack.lm (v1.2.4), CellChat (v2.2.0).

**Predefined Keyword Sets.** To achieve automated functional annotation of the identified PPI modules, a predefined dictionary was compiled based on broad literature consensus regarding ferroptosis mechanisms. The specific keyword sets are as follows:(1) Lipid metabolism keywords:*“phospholipid, lipid, glycerol, glycerolipid, unsaturated fatty acid, fatty acid, membrane lipid, sphingolipid, triglyceride, lipoprotein, fat, cholesterol, cholesterol metabolism, glycolipid, wax, bile acid, steroid, lipogenesis, lipolysis, phospholipase”*; (2) GSH Metabolism Keywords:*“reactive oxygen species, oxidative stress, cysteine, methionine, glutamate, glycine, glutathione, GSH, glutathione metabolism, glutathione peroxidase, homocysteine”*; (3) Iron Metabolism Keywords:*“iron coordination entity transport, iron ion transport, iron, ferroptosis, ferritin, transferrin, heme, hepcidin, ferroportin, iron-sulfur clusters, dcytb, ceruloplasmin, lactoferrin”*.

**Benchmarking against Existing Methods**

To benchmark FerroScore against existing transcriptomic ferroptosis scoring approaches, we compared it with conventional gene set enrichment methods, including ssGSEA and GSVA, as well as ferroptosis-related signature scores, including FPI, FPS, FerrDb_DS, and PCA_FRGscore. For ssGSEA and GSVA, the global reference ferroptosis gene set was retrieved from the FerrDb database, and scores were computed using the GSVA R package with default parameters. For the composite signature scores, FPI, FPS, and FerrDb_DS were implemented by contrasting pro ferroptotic and anti ferroptotic components. Specifically, predefined positive or driver gene sets and negative or suppressor gene sets were scored independently using the ssGSEA algorithm. The final score for each method was then derived by subtracting the suppressor module score from the driver module score:

$$\begin{matrix} \text{FPI=ssGSEA}\left(\text{FPI}_{positive}\right)-\text{ssGSEA}\left(\text{FPI}_{negative}\right), \\ \text{FPS=ssGSEA}\left(\text{FPS}_{pro}\right)-\text{ssGSEA}\left(\text{FPS}_{anti}\right), \\ \text{FerrDb\_DS=ssGSEA}\left(\text{FerrDb}_{drivers}\right)-\text{ssGSEA}\left(\text{FerrDb}_{suppressors}\right). \end{matrix}$$

The FPI positive and negative gene sets were obtained from Liu *et al.* [3], and the FPS pro ferroptotic and anti ferroptotic gene sets were obtained from He *et al.* [4]. For PCA_FRGscore, the expression matrix was subsetted to detected FerrDb_All genes and subjected to principal component analysis after centering and scaling. The score was defined as:

$$\text{PCA}\_\text{FRGscore=PC1+PC2,}$$

with its direction aligned to FerrDb_DS to ensure consistent interpretation. For each method, sample level scores were averaged within each treatment group and compared with experimentally measured cell viability using Pearson correlation coefficients.

**Sensitivity and Robustness Analyses**

**CPM Threshold Sensitivity.** To assess the impact of gene expression filtering stringency, FerroScore was recalculated using a standard relaxed threshold (CPM > 1) instead of the original stringent criterion (CPM > 100). Inclusion of low-abundance transcripts led to substantial perturbations of network topology and altered FerroScore values across experimental conditions (Supplementary Figs. S4–S5, S6A). Correspondingly, correlations with cell viability were markedly attenuated and were no longer statistically significant in either the MEF (*R = −0.42, p = 0.402*) or A549 (*R = −0.06, p = 0.941*) datasets. These results indicate that insufficient expression filtering introduces transcriptional noise that destabilizes network structure and obscures phenotype-associated ferroptosis signals, supporting the use of stringent filtering to preserve the functional core of the ferroptosis network.

**PPI Confidence Threshold Sensitivity.** FerroScore constructs protein–protein interaction networks using a default STRING confidence threshold of 400. To evaluate sensitivity to this parameter, networks were reconstructed using both relaxed (300) and stringent (500) cutoffs. Across all experimental conditions in both the MEF and A549 datasets, the extracted ferroptosis super-node topology, module organization, and resulting FerroScore values remained identical to those obtained under the default setting. These findings demonstrate that FerroScore is robust to moderate variations in PPI confidence thresholds, reflecting the stability of the underlying core ferroptosis interaction network (Supplementary Fig. S6B).

**Topological Metric Normalization.** The five topological metrics integrated into FerroScore ($Q\left(c\right),D\left(c\right),k_{i},BC\left(i\right)$ and $C\left(i\right)$) are empirically constrained in the ferroptosis super-node network, which contains only three metabolic modules with retained self-loops. Under this compressed topology, $k_{i}\in\left\{2,3,4\right\}$, $BC\left(i\right)\in\left\{0,1\right\}$, $C\left(i\right)\in\left\{0,1\right\}$, $D\left(c\right)\in\left(0,2\right]$, and $Q\left(c\right)$ remained close to zero due to finite-size effects, preventing any single metric from dominating the composite score. To assess potential scale effects, $D\left(c\right)$ and $k_{i}$ were min–max normalized ($D\left(c\right)/2$ and $\left(k_{i}-2\right)/2$), while $Q\left(c\right)$,$BC\left(i\right)$, and $C\left(i\right)$ were left unchanged. FerroScore was recalculated for both the MEF and A549 datasets. Relative rankings of experimental conditions were fully preserved after normalization in both datasets, indicating strong robustness to metric rescaling. Although statistical significance was reduced in MEF (*p = 0.256*), likely reflecting variance compression under small sample size, correlations in A549 remained significant (*p = 0.026*). Raw and normalized metrics, along with corresponding FerroScore values, are provided in Supplementary Tables S1–S2, and correlations with cell viability are shown in Supplementary Fig. S6C.

**Reference Background Sensitivity.** FerroScore is initialized from DEGs defined relative to a control group; consequently, changes in the reference background may affect PPI reconstruction, module detection, ferroptosis super-node annotation, and the final score. To evaluate this property, we reran the complete FerroScore pipeline in the MEF and A549 datasets using two schemes that perturbed the reference background. In the original matched control setting, each treatment was compared with its paired control group with two replicates: MEF AA/Cys used mRNA_NT_1 + mRNA_NT_2, MEF CysAm/sh-CTNS used NT_1 + NT_2, and A549 IKE/RSL3 and KO_IKE/KO_RSL3 used NC_DMSO_1 + NC_DMSO_2 and KO_DMSO_1 + KO_DMSO_2, respectively. For combined MEF perturbations, shared DEGs were defined as the intersection of DEGs from the corresponding individual treatments. In the matched control leave-one-out analysis, each paired control replicate was used separately while treated samples were kept unchanged; for example, MEF AA was analyzed as mRNA_NT_1 versus mRNA_AA_1 + mRNA_AA_2 and mRNA_NT_2 versus mRNA_AA_1 + mRNA_AA_2, whereas A549 IKE was analyzed as NC_DMSO_1 versus NC_IKE_1 + NC_IKE_2 and NC_DMSO_2 versus NC_IKE_1 + NC_IKE_2. In the pooled control stress test, broader basal references were constructed by combining mRNA_NT_1 + mRNA_NT_2 + NT_1 + NT_2 for MEF and NC_DMSO_1 + NC_DMSO_2 + KO_DMSO_1 + KO_DMSO_2 for A549. Detailed DEG counts, PPI network sizes, recovered modules, and FerroScore values are provided in Supplementary Table S3. This analysis showed that A549 retained identifiable ferroptosis core modules and estimable FerroScore values across the tested reference settings, indicating that its module structure and scores were relatively stable to reference perturbation. In contrast, MEF exhibited stronger condition dependent sensitivity. This sensitivity was most apparent under pooled controls, where combining distinct NT backgrounds altered the DEG and PPI network composition and reduced the recovery of core modules related to lipid, iron, or GSH metabolism in several MEF conditions. These results support FerroScore as a relative metric that is sensitive to the reference background and highlight the importance of biologically matched controls.

**Statistical Analysis of Bulk RNA‑seq Data**

**Locally Weighted Regression.** The LOESS method was applied to capture the nonlinear relationship between ferroptosis score and patient survival time in the TCGA‑PAAD cohort. The smoothing parameter *span* was optimized via grid search based on the generalized cross-validation criterion (set range 0.1–1.0, step size 0.05), which identified *span* = 0.45 as the optimal value. Using this parameter, the LOESS model was fitted and its 95% confidence interval was computed, yielding the final optimized regression curve.

**Derivation of the Composite Function Model.** Based on the nonlinear relationship between ferroptosis score and survival time identified by LOESS regression, we adopted a piecewise function strategy to construct the outer layer of the composite model. Specifically, two inflection points (ferroptosis scores = 0.45 and 0.77) were identified according to the curve morphology, and the data were divided into three intervals: For scores $\leq0.45$, linear regression modeled the negative association between ferroptosis score and survival time; For scores between 0.45–0.77, nonlinear least squares were applied to fit a quadratic function, capturing the typical U-shaped relationship; and for scores > 0.77, linear regression characterized the gradual increase in survival time with rising ferroptosis scores. All segment models were parameterized using least squares criterion. To further dissect the regulatory effects of signaling pathways on ferroptosis activity, we developed the inner layer of the composite model using a multi-model integration strategy. First, based on the MSigDB Hallmark gene sets, TPM expression data were converted into activity scores for 50 pathways using the GSVA algorithm. The LightGBM model was then applied for preliminary screening of important pathways, followed by the use of elastic net regression ($\alpha=1$, $\lambda=0.045$), to construct a biologically interpretable linear model. The model was trained with 10-fold cross‑validation, and the dataset was split 8:2 into training and test sets. Preprocessing removed low-variance pathways (*frequency ratio* $>95/5$) and highly correlated pathways (*r*> 0.9). Key pathways and their regulatory directions were ultimately determined via elastic net regression.

**Prognostic Independence Analysis.** To rigorously evaluate whether the prognostic value of the FerroScore is independent of tumor stage, a stratified survival analysis was performed focusing on the early-stage (Stage I & II) sub-cohort (*n=124*) from the TCGA-PAAD dataset. Advanced-stage patients (Stage III & IV) were excluded from this analysis due to a limited sample size, which precludes robust multivariable adjustment. PFI was selected as the primary clinical endpoint. In contrast to overall survival, which may be confounded by postoperative complications and non-cancer-related mortality (particularly in early-stage pancreatic cancer), PFI more directly reflects intrinsic tumor recurrence risk and biological aggressiveness. Patients were dichotomized into high- and low-FerroScore groups using the optimal cutoff determined by maximally selected rank statistics. Survival differences were assessed using Kaplan-Meier curves and the log-rank test. As shown in Fig. 3E, patients with high FerroScore exhibited significantly prolonged PFI compared with the low-score group (*p = 0.044*). This result demonstrates that FerroScore captures prognostic information distinct from anatomical staging, effectively stratifying recurrence risk even within a clinically homogeneous early-stage cohort.

**Acquisition of High-Frequency Consensus Differentially Expressed Genes.** To investigate the evolution of ferroptosis-related molecular mechanisms across tumor stages, stage-specific differential expression analysis based on the TCGA-PAAD cohort was performed. Significantly DEGs (*adjusted p-value* < 0.05, *|log2FC|* > 0.5) were identified separately for stages I–IV. High-frequency consensus genes were defined as those detected in $\geq50$% of patients within the same stage. The top 20 high-frequency genes from each stage were selected for visualization, with grouped bar charts displaying their expression change directions, and row-normalized heatmaps showing expression patterns across the four stages. Hierarchical clustering of the heatmap was performed using Euclidean distance and complete linkage to reveal stage-specific expression characteristics.

**Statistical Analysis of scRNA‑seq Data**

**Cell Annotation of Immune and Non‑Immune Cells.** Based on pancreatic cancer scRNA‑seq data, a hierarchical annotation strategy was employed to systematically identify immune cell subpopulations. First, unsupervised clustering was performed on the preprocessed data using Seurat (*resolution*= 2.0), and major immune cell types were identified via canonical marker genes: T cells (CD3D, CD3E, CD8A, CD2, CD7, CD52, CD3G), B cells (CD19, CD79A, MS4A1, CD79B, VPREB3), Macrophages (CD14, CD68, CD163, LYZ, C1QC, CD4, KLF2), DC cells (CLEC10A, SELL, CD4, CLEC4C), Monocytes (CD14, FCN1, S100A8, FCGR3A), NK cells (KLRD1, NKG7, FGFBP2, TRDC), and Mast cells (KIT, CPA3, TPSAB1, TPSB2, MS4A2). Referring to the cell annotation approach by Kim et al. [5], major cell populations were further subdivided: T cells into CD8⁺ Tem/Teff, Tcm, Trm, Trm_exhausted, and CD4⁺ Tem/Teff, Tcm, Treg; B cells into Naïve B, Memory B, and Plasma cells; and DC cells into cDC1, cDC2, and pDC. Ultimately, this yielded 20 finely annotated immune cell subtypes, visualized via UMAP. For non‑immune cells, unsupervised clustering (*resolution*= 1.0) and canonical markers were first used to identify five main cell types: Epithelial cells (KRT8, KRT18, KRT19, CDH1, EPCAM, CLDN4), Endothelial cells (PECAM1, CDH5, CD34, VWF), Fibroblasts (FAP, PDGFRA, FGF7, LUM, DCN), Stellate cells (RGS5), and Schwann cells (SOX10). Subtypes were then defined based on intrinsic functional profiles. DEGs for each subtype were identified using the Wilcoxon rank‑sum test (*min.pct* = 0.25, *logfc.threshold* = 1), and functional characteristics were validated by GO biological process enrichment. Based on marker expression and differential gene patterns, subtype subdivisions were obtained for Epithelial, Endothelial, Fibroblast, and Stellate cells. Epithelial cells (11771 cells) were identified with 7 subtypes: EMTEp, metEp, imEp, pEp, sEp, ciliumEp, and sarcomatoidEp; Endothelial cells (2284 cells) were divided into 6 subtypes: lEn, vEn_Artery, vEn_Vein, vEn_Capillary, iEn, and EMTEn; Fibroblast cells (11725 cells) were identified with 5 subtypes: mCAF, iCAF, hspCAF, dCAF, and nCAF; Stellate cells (2105 cells) were classified into 6 subtypes: fPSC, metPSC, iPSC, imPSC, nPSC, and cPSC. The above process resulted in a functionally resolved atlas of 25 non‑immune cell subtypes. Dot plots of canonical marker genes for all immune and non-immune cell types are provided in Supplementary Fig. S9.

**Construction of Transcription Factor–Target Gene Regulatory Networks.** The TF–targeted gene regulatory network was constructed in three steps. First, key driver genes were identified. Candidate driver genes were selected by integrating multiple gene sets, including the core genes (top 20%) from significantly enriched GSEA pathways (*FDR* < 0.05 and *|NES|* > 1.5), as well as known ferroptosis-related genes from the FerrDb database. DEGs (*P* < 0.05 and *|logFC|* > 1) belonging to any of these sets were considered as candidate drivers. A PPI network was built from the STRING database (*confidence threshold* $\geq400$), and six topological features were computed for each node: *degree*, *betweenness*, *hub_score*, *closeness*, *eigen_centrality*, and *page_rank*. After Z‑score normalization of each metric, PCA was applied to integrate these multidimensional features. The first principal component was served as a composite importance score, and the top 10 genes ranked by this score were defined as key driver genes. Their multidimensional topological profiles were visualized as radar charts. Next, TFs for each key driver gene were predicted. Based on the TRRUST database, all TFs known to regulate the key driver genes were extracted to construct an initial regulatory network. Four centrality metrics (*degree*, *closeness*, *eigen_centrality*, and *hub_score*) were computed for each TF node. After Z‑score normalization, PCA was again used to integrate these metrics, and the first principal component was served as a regulatory importance score. The top 10 TFs based on this score were designated as key regulators. Finally, the core regulatory network was constructed by selecting the key TFs and their directly regulated driver genes. In the final network visualization, node size was proportional to the PCA-based importance score, and node color differentiated TFs from target genes.

**Construction of the “Cell–Gene–Signal” Autocrine and Paracrine Ferroptosis Network.** Seven cell types with high ferroptosis activity (Macrophage, CD8 Tcm, NK, nCAF, iPSC, EMTEn, imEp) were selected as cell nodes. Gene nodes were defined by the overlap between known ferroptosis-related genes from FerrDB and cell-type-specific DEGs. Signal nodes were selected from all signaling pathways that were significantly activated in high-ferroptosis cells during intercellular communication, as identified in prior analysis. Connections between nodes were established according to the following rules: cell-gene edges required both significant expression in the specific cell type and an established association with ferroptosis; gene-pathway edges were assigned based on the top 20% of pathways by relevance from GeneCards and STRING databases; and pathway–cell edges were derived from the cell–cell communication probability matrix. An edge was created if target cells exhibited autocrine signaling or had communication probabilities >0 with other cell types in specific pathways. This integrated approach produced a "Cell–Gene–Signal" network that captured both autocrine and paracrine regulatory mechanisms in ferroptosis.

**Supplementary Figures**


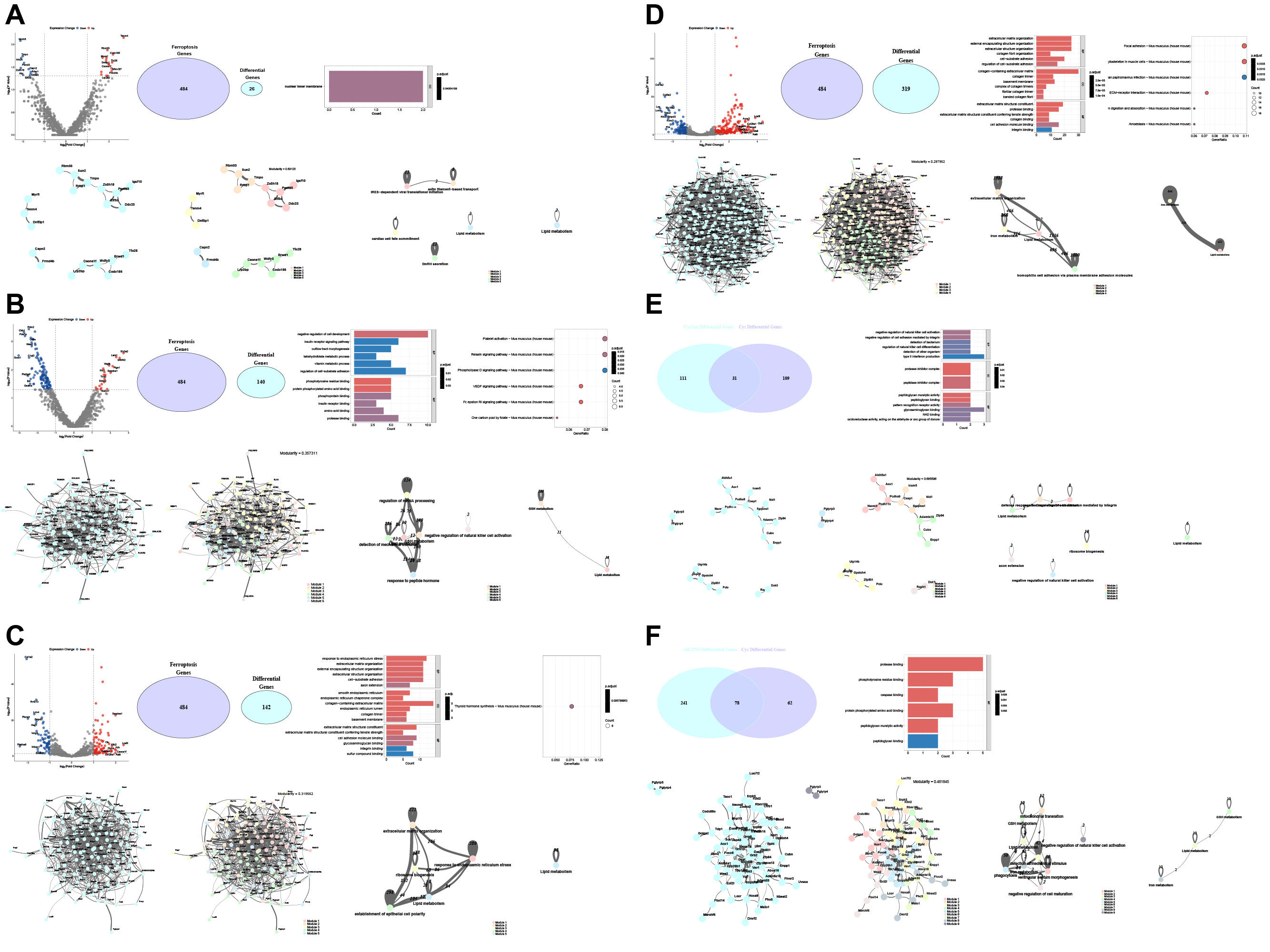


**Fig. S1. Assessment of the Ferroptosis score in the MEF Cell Line.** **(A–F)** Detailed analytical results of FerroScore under six experimental groups: **(A)** AA treatment, **(B)** Cys treatment, **(C)** CysAm treatment, **(D)** sh‑CTNS treatment, **(E)** Cys + CysAm combination treatment, and **(F)** Cys + sh‑CTNS combination treatment. Panel includes a volcano plot of DEGs, a Venn diagram, preliminary GO and KEGG functional analyses, the PPI network, the modular PPI network, the super‑node network, and the final extracted ferroptosis‑related network. AA: full amino acid starvation, Cys: cysteine starvation, CysAm: cysteamine treatment, sh‑CTNS: sh‑CTNS transfection.


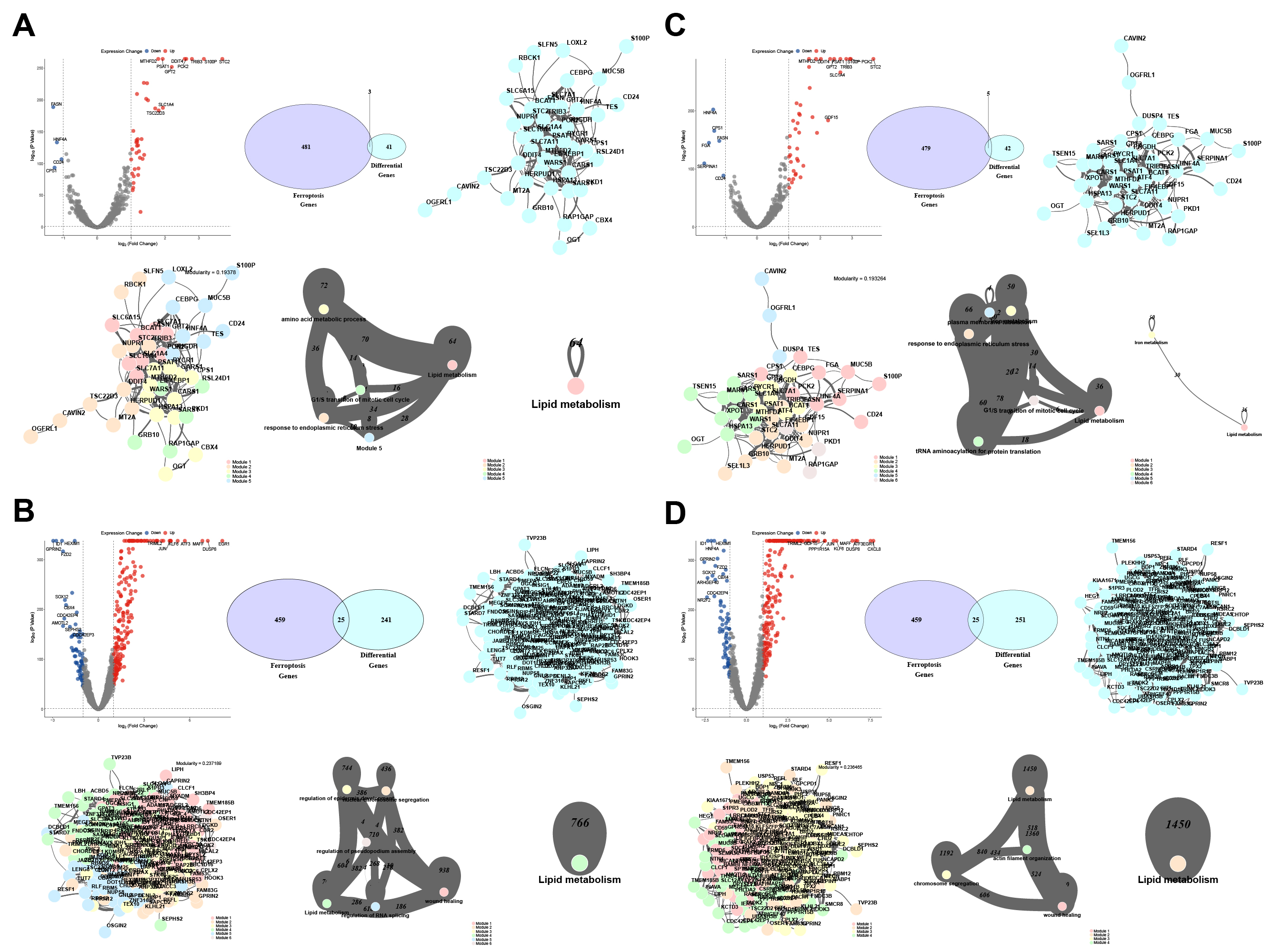


**Fig. S2. Assessment of the Ferroptosis score in the A549 Cell Line.** **(A–D)** Detailed analytical results of FerroScore under four experimental groups: **(A)** IKE treatment, **(B)** RSL3 treatment, **(C)** KO_IKE treatment, and **(D)** KO_RSL3 treatment. Panel includes a volcano plot of DEGs, a Venn diagram, a PPI network, a modular PPI network, a super-node network, and the final extracted ferroptosis-related network.


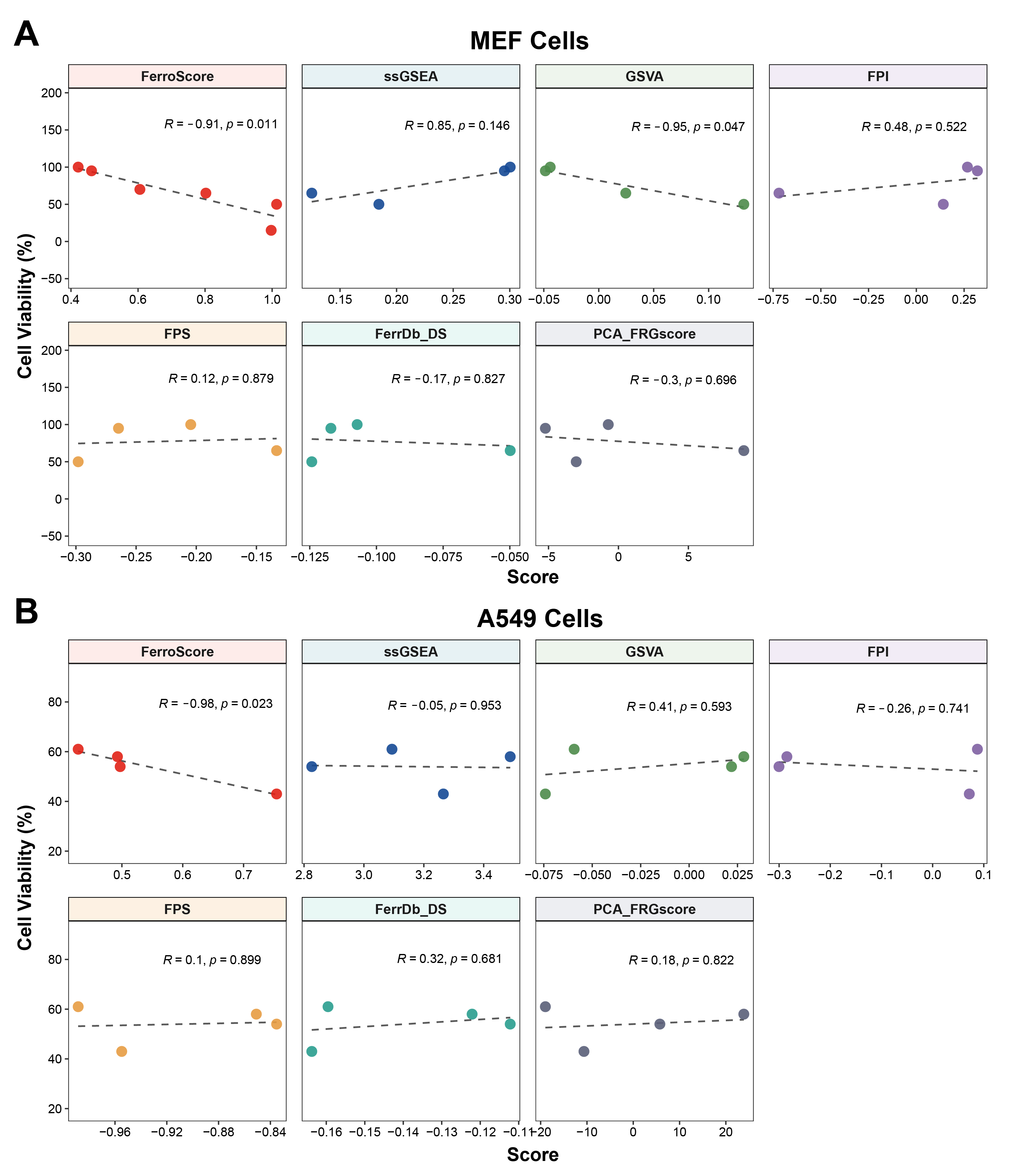


**Fig. S3. Comprehensive benchmarking of FerroScore against existing transcriptomic ferroptosis scoring methods.** **(A–B)** Detailed scatter plots showing the correlation between various computational scores and experimentally measured cell viability in **(A)** MEF cells and **(B)** A549 cells. The evaluated methods include FerroScore, conventional gene set enrichment approaches (ssGSEA and GSVA), and specialized ferroptosis signature scores (FPI, FPS, FerrDb_DS, and PCA_FRGscore). Each point represents an experimental group. Dashed lines indicate linear regression fits. Pearson correlation coefficients (*R*) and nominal *p* values are shown in each panel.


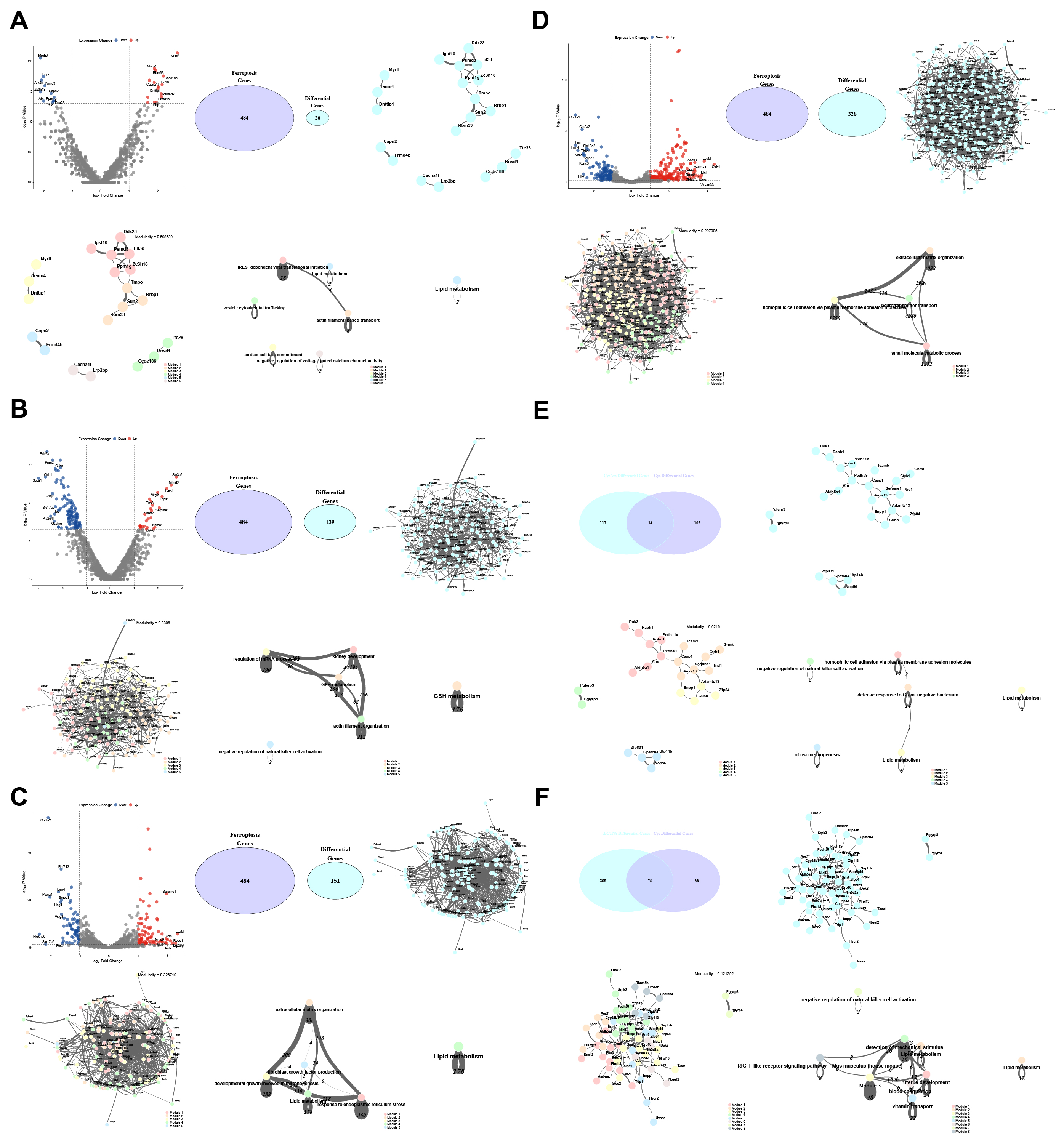


**Fig. S4. Assessment of FerroScore network topology under relaxed gene filtering stringency (CPM > 1) in the MEF Cell Line.** **(A–F)** Detailed analytical results of FerroScore applied with a relaxed expression threshold (CPM > 1) across six experimental groups: **(A)** AA treatment, **(B)** Cys treatment, **(C)** CysAm treatment, **(D)** sh‑CTNS treatment, **(E)** Cys + CysAm combination treatment, and **(F)** Cys + sh‑CTNS combination treatment. Similar to **Fig. S1**, each panel includes a volcano plot of DEGs, a Venn diagram, the PPI network, the modular PPI network, the super‑node network, and the final extracted ferroptosis‑related network.


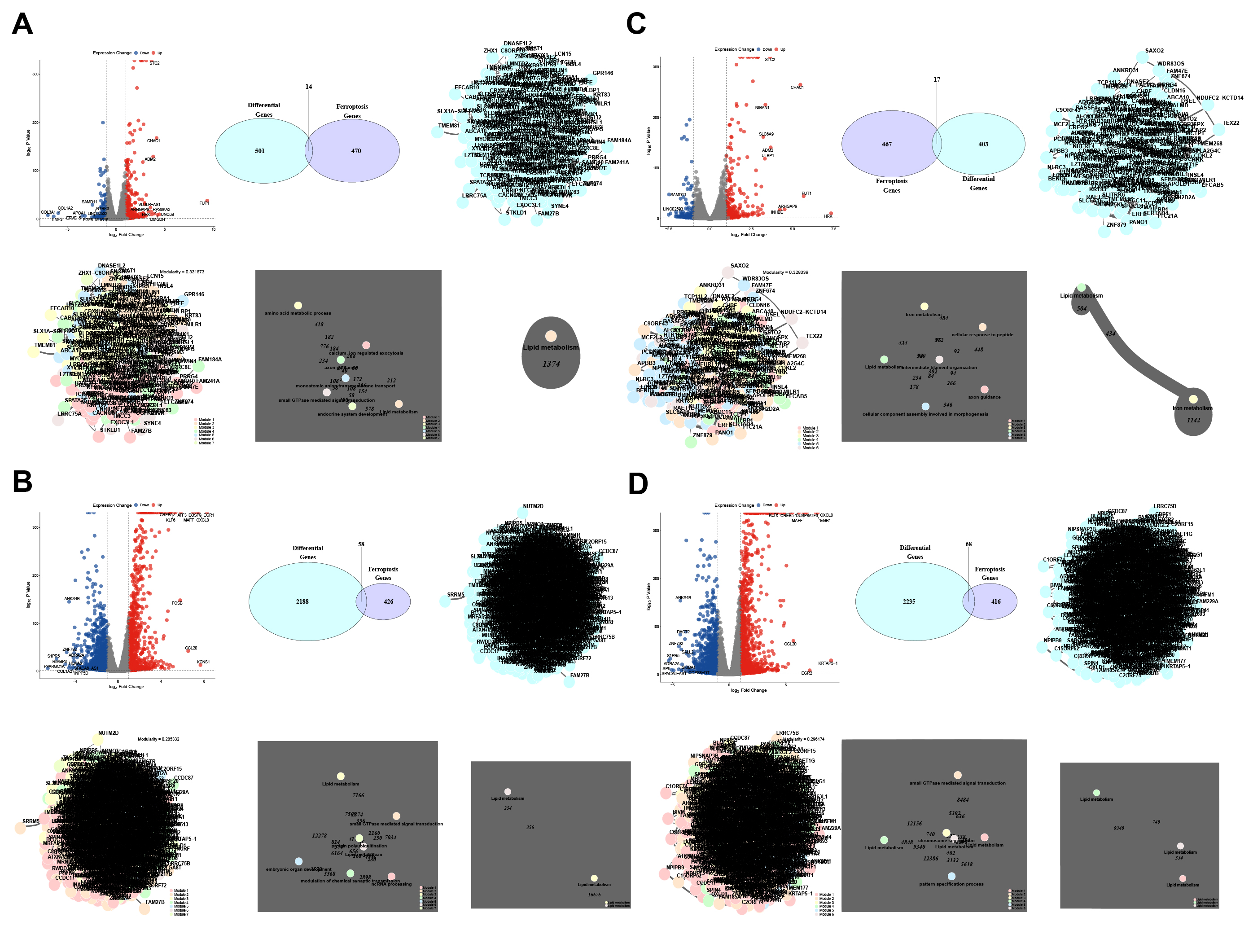


**Fig. S5. Assessment of FerroScore network topology under relaxed gene filtering stringency (CPM > 1) in the A549 Cell Line.** **(A–D)** Detailed analytical results of FerroScore applied with a relaxed expression threshold (CPM > 1) under four experimental groups: **(A)** IKE treatment, **(B)** RSL3 treatment, **(C)** KO_IKE treatment, and **(D)** KO_RSL3 treatment. Similar to **Fig. S2**, each panel includes a volcano plot of DEGs, a Venn diagram, a PPI network, a modular PPI network, a super-node network, and the final extracted ferroptosis-related network.


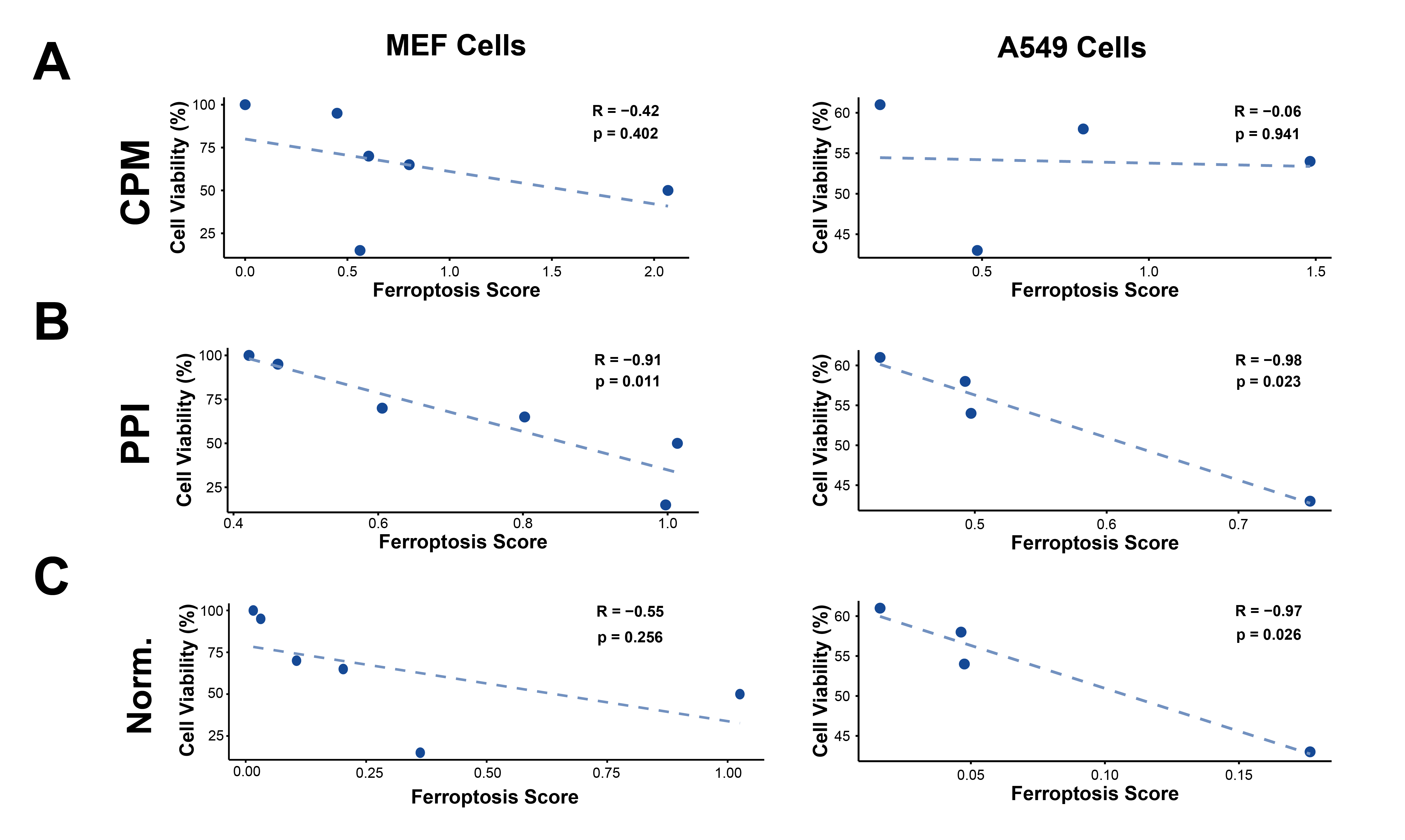


**Fig. S6. Sensitivity and Robustness Analysis of FerroScore in MEF and A549 cell lines.** Correlation analysis between FerroScore and cell viability was performed under three computational conditions: **(A)** varying gene expression filtering thresholds (CPM); **(B)** different PPI network confidence scores; and **(C)** with or without topological metric normalization. Left panels: MEF cells; Right panels: A549 cells. *R* and *p* values represent the Pearson correlation coefficient and statistical significance, respectively.


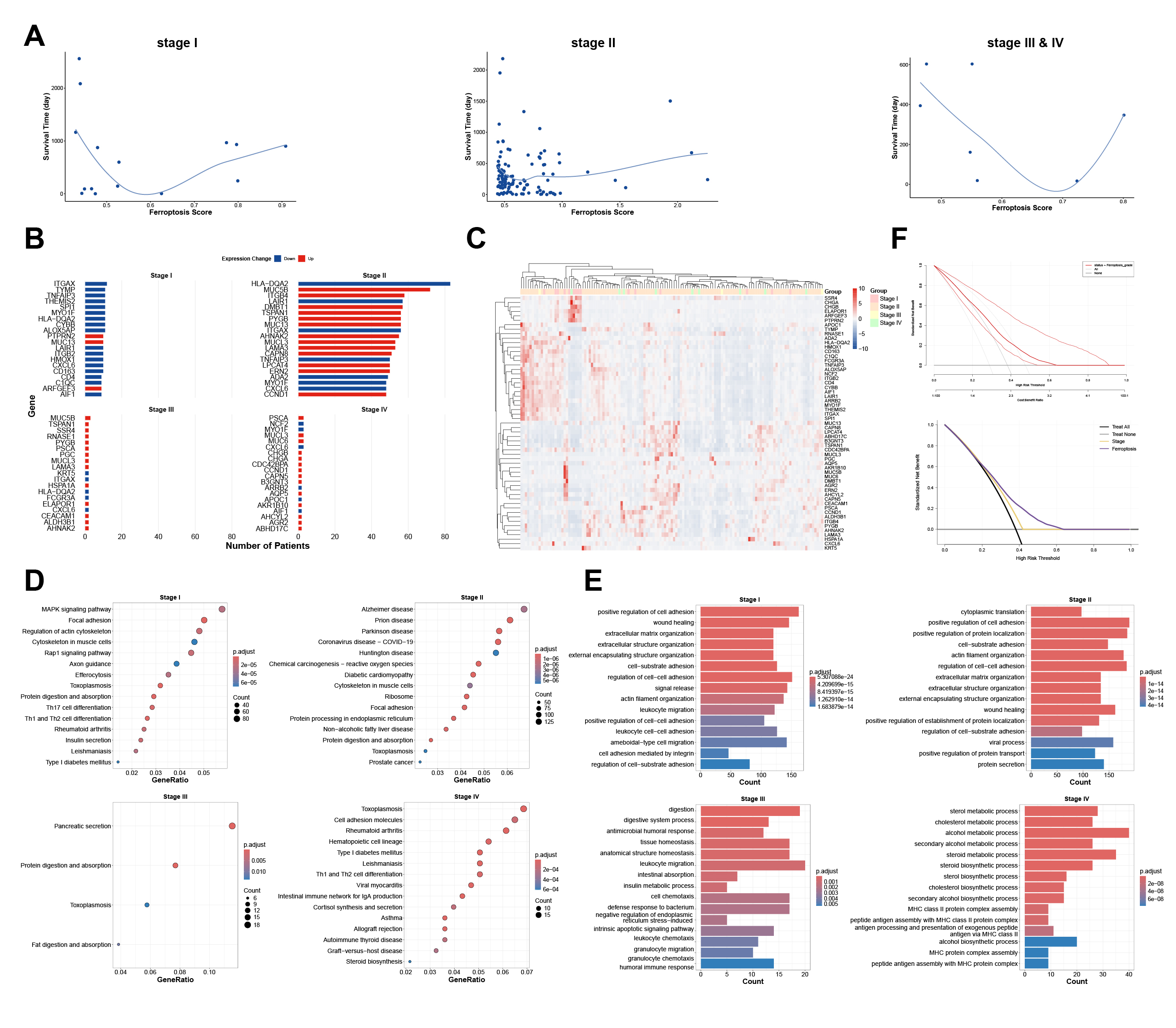


**Fig. S7. Tumor Stage-Specific Ferroptosis Characteristics and the Clinical Value of the Ferroptosis Index. (A)** Stage-specific empirical curves of ferroptosis score versus survival time. **(B)** Bar plot showing the distribution of high-frequency consensus DEGs across tumor stages. Red and blue bars represent up- and down-regulated genes, respectively. **(C)** Heatmap displaying expression patterns of high-frequency consensus DEGs across tumor stages. **(D–E)** Results of KEGG pathway enrichment analysis **(D)** and GO term enrichment analysis **(E)** for each tumor stage. **(F)** Decision curve analysis evaluating the clinical predictive performance of the ferroptosis index. The upper panel shows the decision curve of the ferroptosis index as a standalone predictor (thick red curve), with the shaded area representing its 95% confidence interval (thin red curves). The lower panel compares integrated prediction models, including tumor stage (yellow curve) and the ferroptosis index (purple curve), with "treat all" (black curve) and "treat none" (gray curve) as reference strategies.


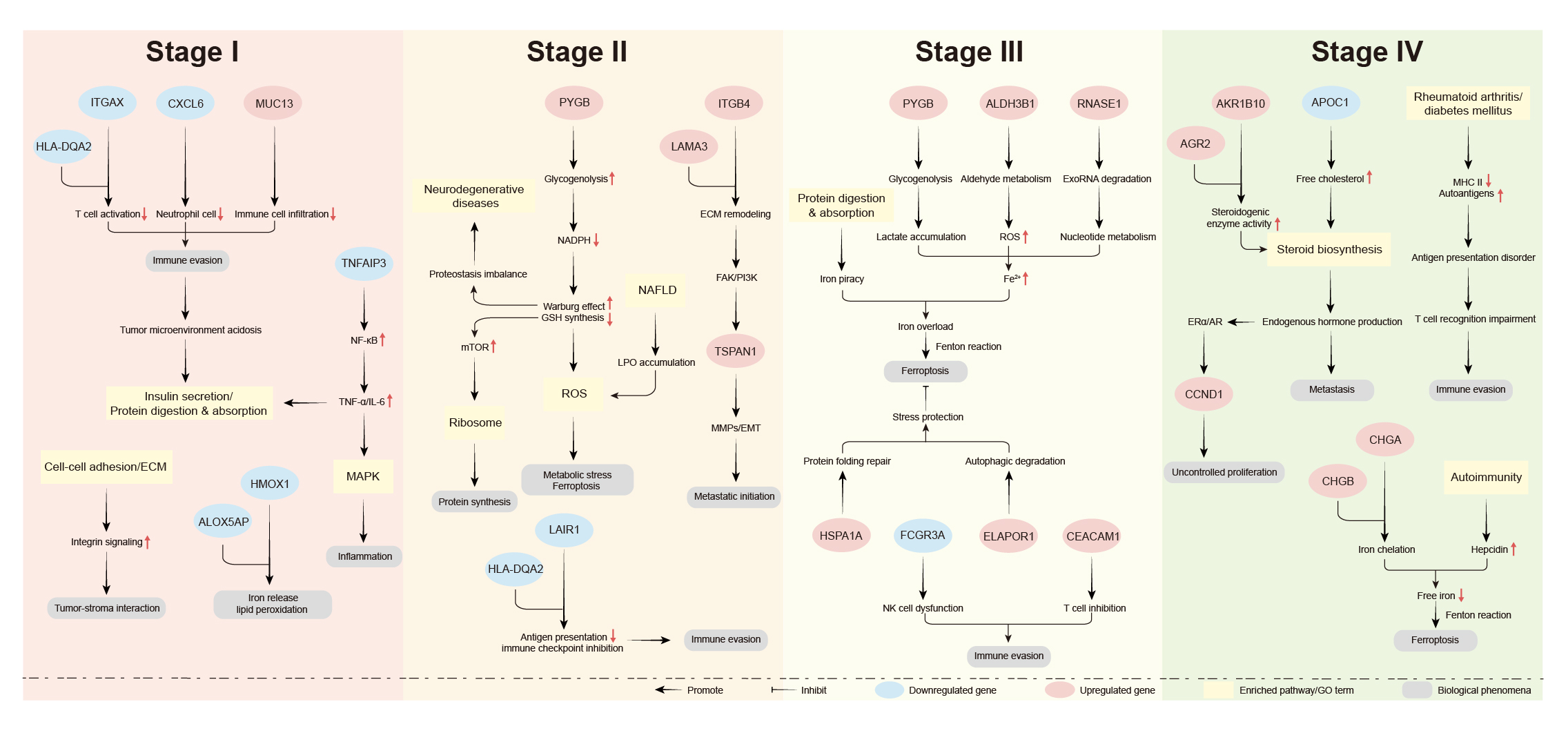


**Fig. S8. Ferroptosis Mechanism Map Across Pancreatic Cancer Stages I–IV.** Based on DEGs, KEGG and GO enrichment analyses from **Fig. S7**, an integrated ferroptosis regulatory mechanism map was constructed for different tumor stages. In the diagram, blue ovals represent down-regulated genes, red ovals represent up-regulated genes, yellow rectangles indicate significantly enriched pathways or GO terms, and gray boxes denote relevant biological processes or phenotypes. Solid arrows indicate activation or promotion, and flat-headed arrows represent inhibition or suppression.


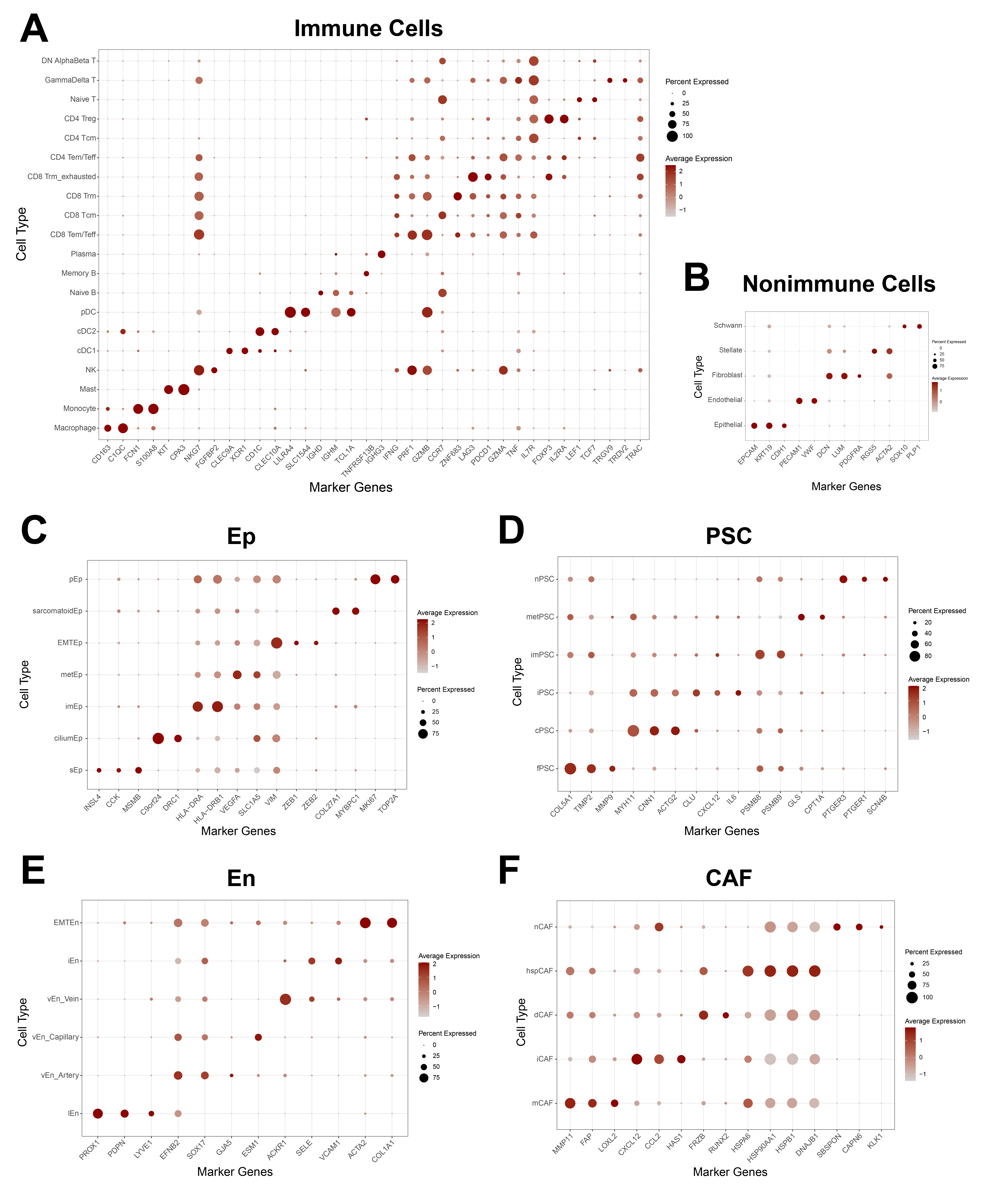


**Fig. S9. Canonical Marker Gene Expression across Immune and Non-immune Cell Types. (A)** Dot plot of marker genes for immune cell types. **(B)** Dot plot of marker genes for major non-immune cell types. **(C–F)** Dot plots of marker genes for non-immune cell subtypes, including Epithelial **(C)**, Stellate **(D)**, Endothelial **(E)**, and Fibroblast **(F)** cells. Ep: Epithelial, PSC: Stellate, En: Endothelial, CAF: Fibroblast.


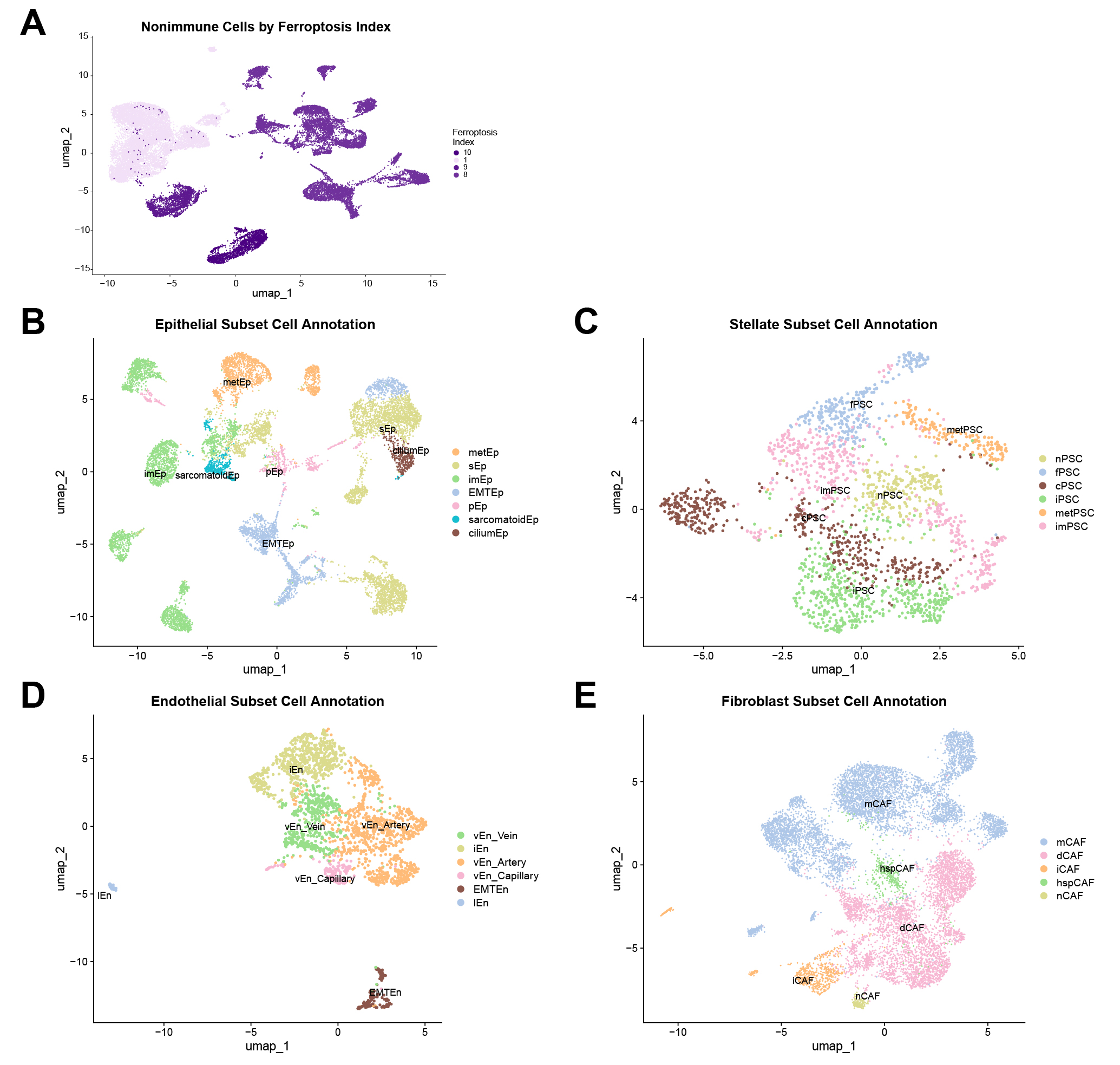


**Fig. S10. Subtype Analysis of Non-immune Cell Populations.** **(A)** UMAP visualization of ferroptosis index across major non-immune cell types, including Epithelial, Endothelial, Fibroblast, Stellate, and Schwann cells. **(B–E)** Subtype-resolved atlas of Epithelial **(B)**, Stellate **(C)**, Endothelial **(D)**, and Fibroblast **(E)** cells. fPSC: fibrotic PSC, metPSC: metabolic PSC, iPSC: inflammatory PSC, imPSC: immunomodulatory PSC, nPSC: neural-regulatory PSC, cPSC: contractile PSC, mCAF: matrix CAF, iCAF: inflammatory CAF, hspCAF: heat shock protein CAF, dCAF: developmental and differentiation-capable CAF, nCAF: neural-modulatory CAF, lEn: lymphatic Endothelial, vEn_Artery: Artery-like vascular Endothelial, vEn_Vein: Vein-like vascular Endothelial, vEn_Capillary: Capillary vascular Endothelial, iEn: inflammatory Endothelial, EMTEn: EMT-like Endothelial, EMTEp: EMT-like Epithelial, metEp: metabolic Epithelial, imEp: immunomodulatory Epithelial, pEp: proliferating Epithelial, sEp: secretory Epithelial.


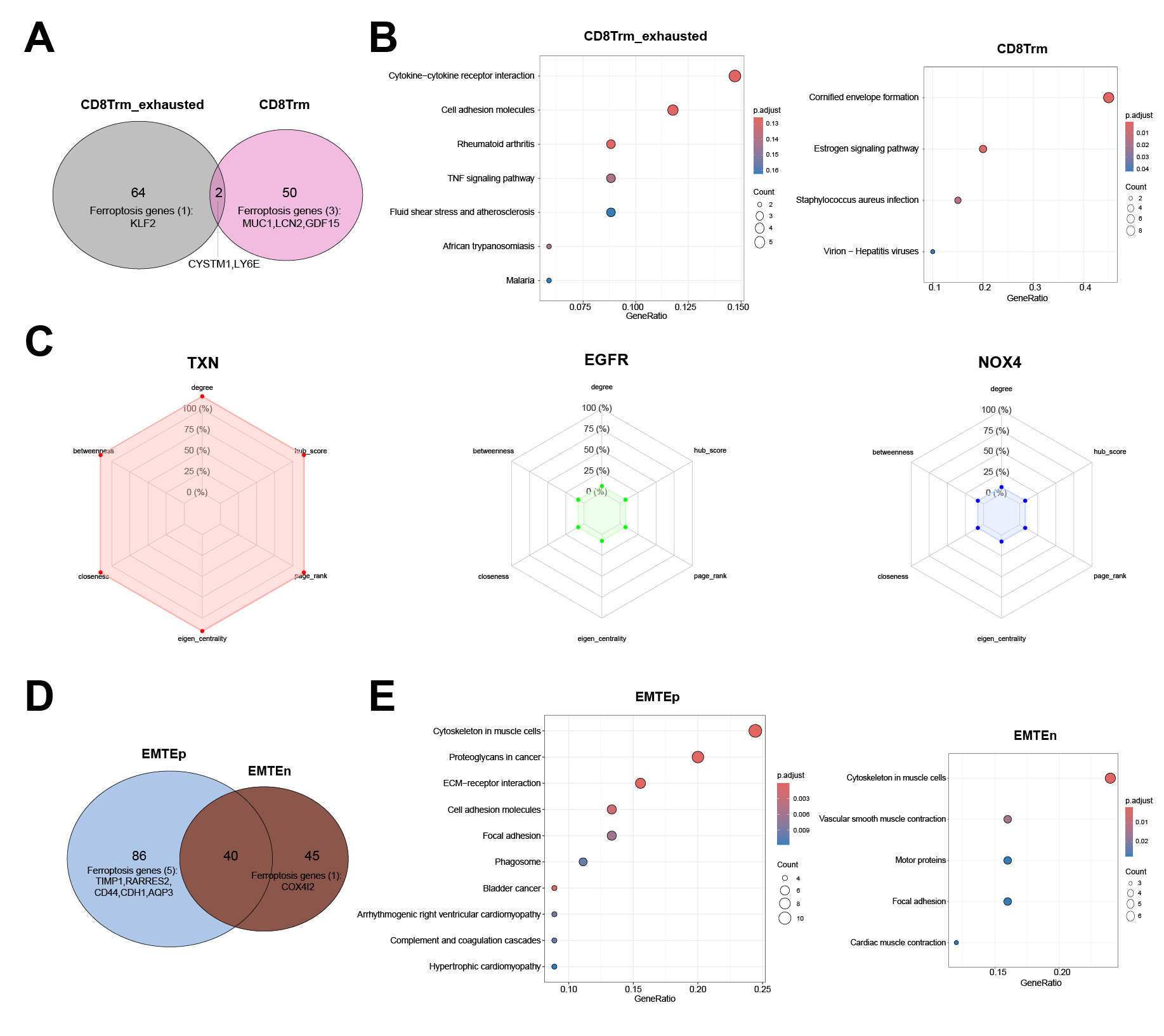


**Fig. S11. Mechanistic Analysis of High-Ferroptosis Stromal Cell Subtypes in the Tumor Microenvironment. (A)** Subtype-specific and shared DEGs, along with known ferroptosis-related genes, in CD8 Trm_exhausted versus CD8 Trm cells. **(B)** KEGG pathway enrichment analysis of subtype-specific genes in CD8 Trm_exhausted and CD8 Trm cells. **(C)** Radar chart of key ferroptosis driver genes TXN, EGFR, and NOX4 in nCAF cells. **(D)** Subtype-specific and shared DEGs, and known ferroptosis-related genes, in EMTEp versus EMTEn cells. **(E)** KEGG pathway enrichment analysis of subtype-specific genes in EMTEp and EMTEn cells.


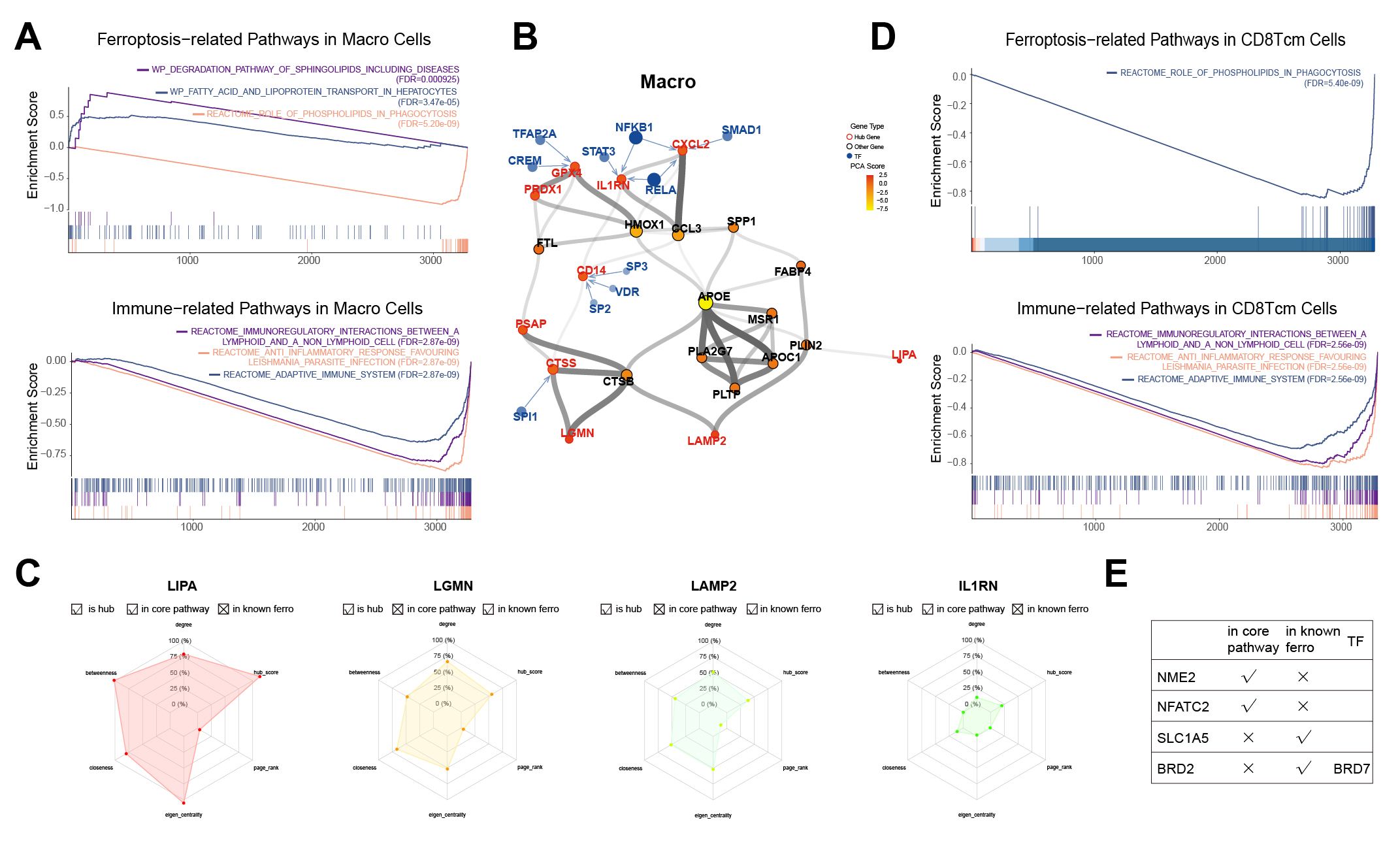


**Fig. S12. Ferroptosis-Mediated Remodeling of Immune Cell Function.** **(A)** GSEA of ferroptosis- and immune-related pathways in Macrophages. **(B)** TF–target gene regulatory network in Macrophages. **(C)** Radar chart of key ferroptosis driver genes LIPA, LGMN, LAMP2, and IL1RN in Macrophages. **(D)** GSEA of ferroptosis- and immune-related pathways in CD8 Tcm cells. **(E)** Key ferroptosis driver genes and their corresponding TFs in CD8 Tcm cells.


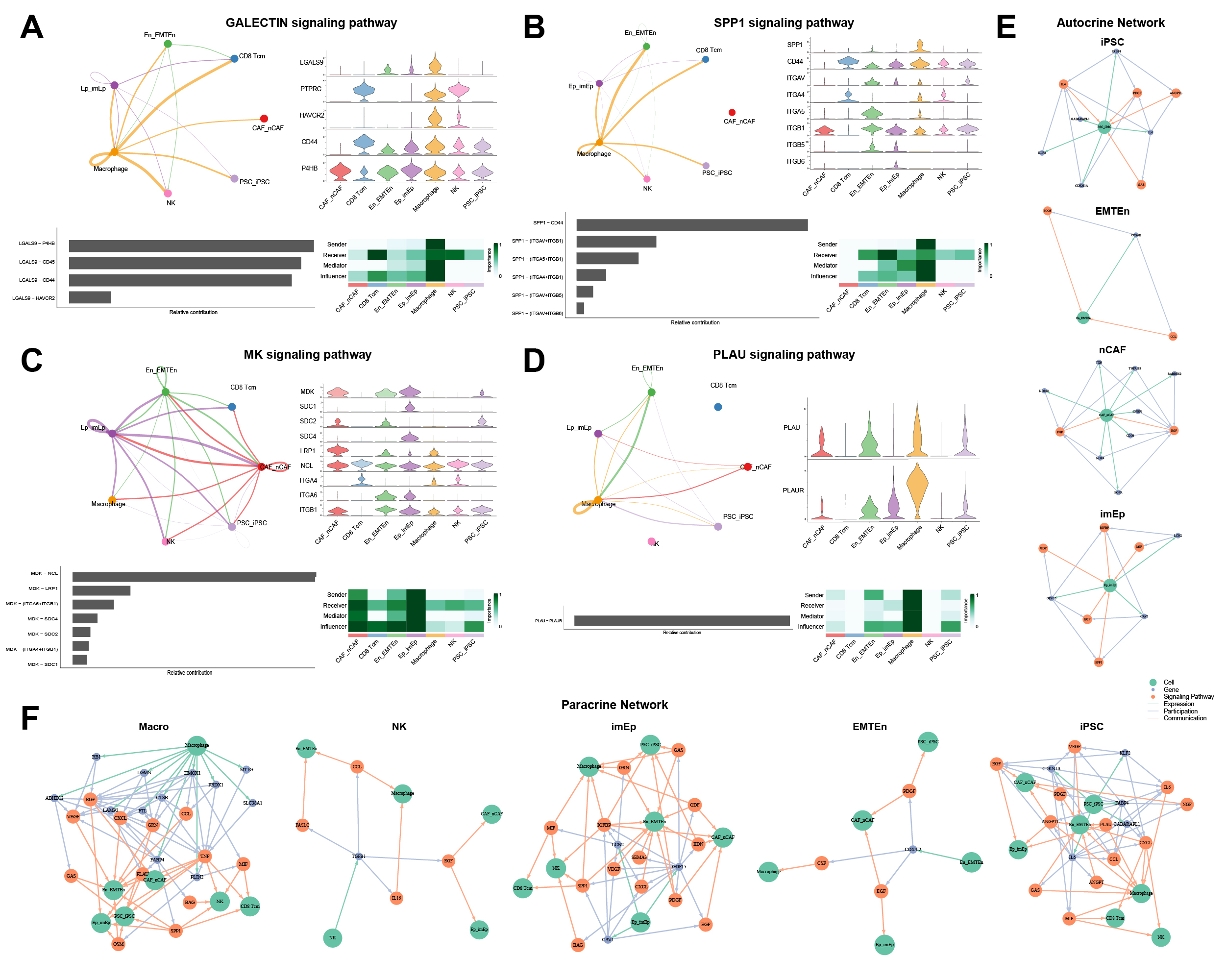


**Fig. S13. Regulatory Mechanisms of Ferroptosis and Intercellular Communication in the Tumor Immune Microenvironment.** **(A–D)** Analysis of the GALECTIN **(A)**, SPP1 **(B)**, MK **(C)**, and PLAU **(D)** signaling pathways in high-ferroptosis cell subtypes, including the cell–cell communication network structure, gene expression patterns, ligand–receptor pair contributions, and functional roles of cell subtypes (e.g., sender, receiver, mediator, or influencer cells). **(E–F)** Ferroptosis "Cell–Gene–Signal" autocrine **(E)** and paracrine **(F)** regulatory networks in high-ferroptosis cell subtypes.


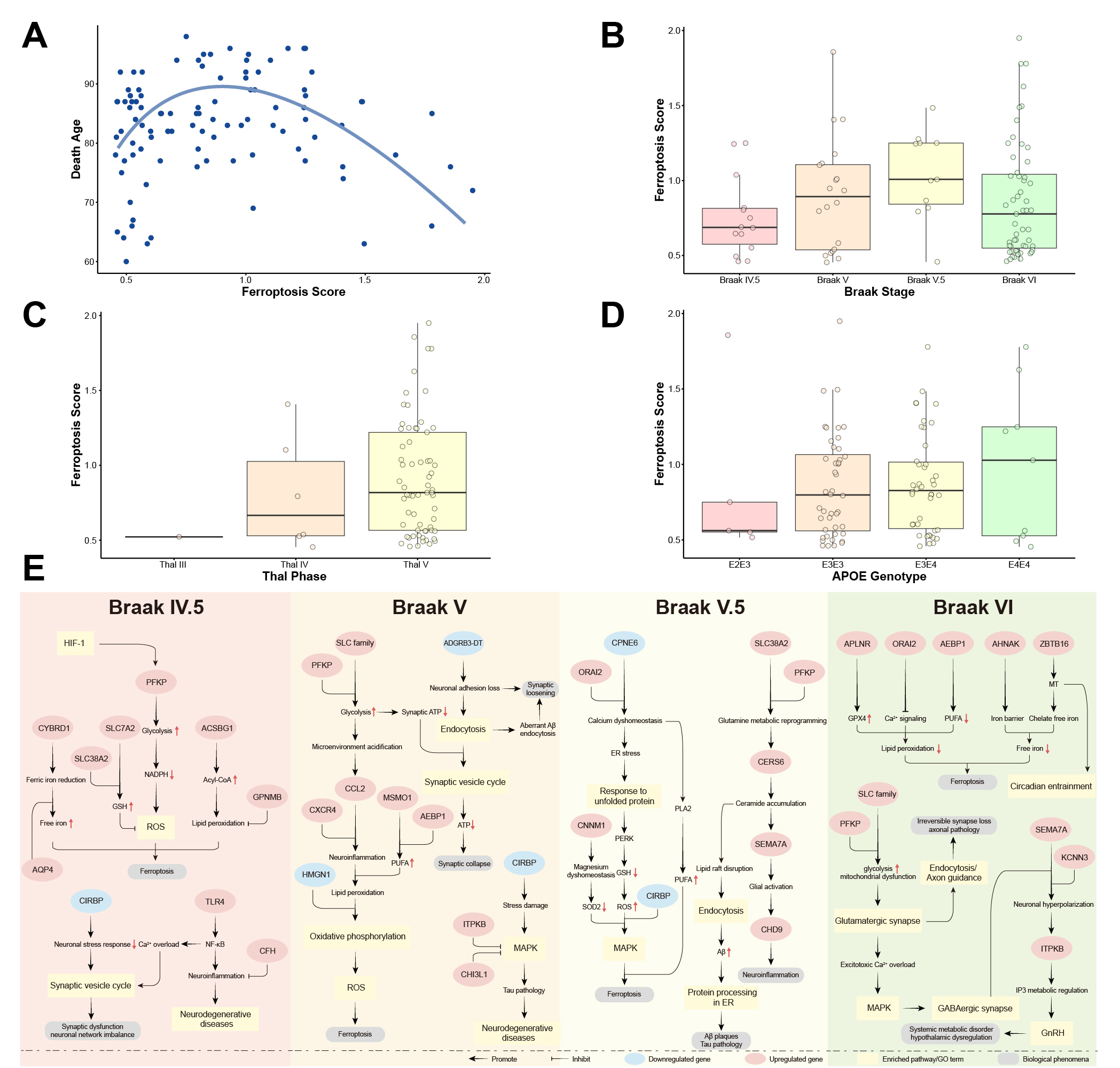


**Fig. S14. Application of FerroScore in Alzheimer's Disease and Investigation of Its Underlying Mechanisms.** **(A)** Empirical curve of ferroptosis score and age at death in AD patients. **(B–D)** Box plot association analyses between ferroptosis scores and Braak stage **(B)**, Thal stage **(C)**, and APOE genotype **(D)**. **(E)** Ferroptosis mechanism map across Braak stages IV.5 to VI in AD.


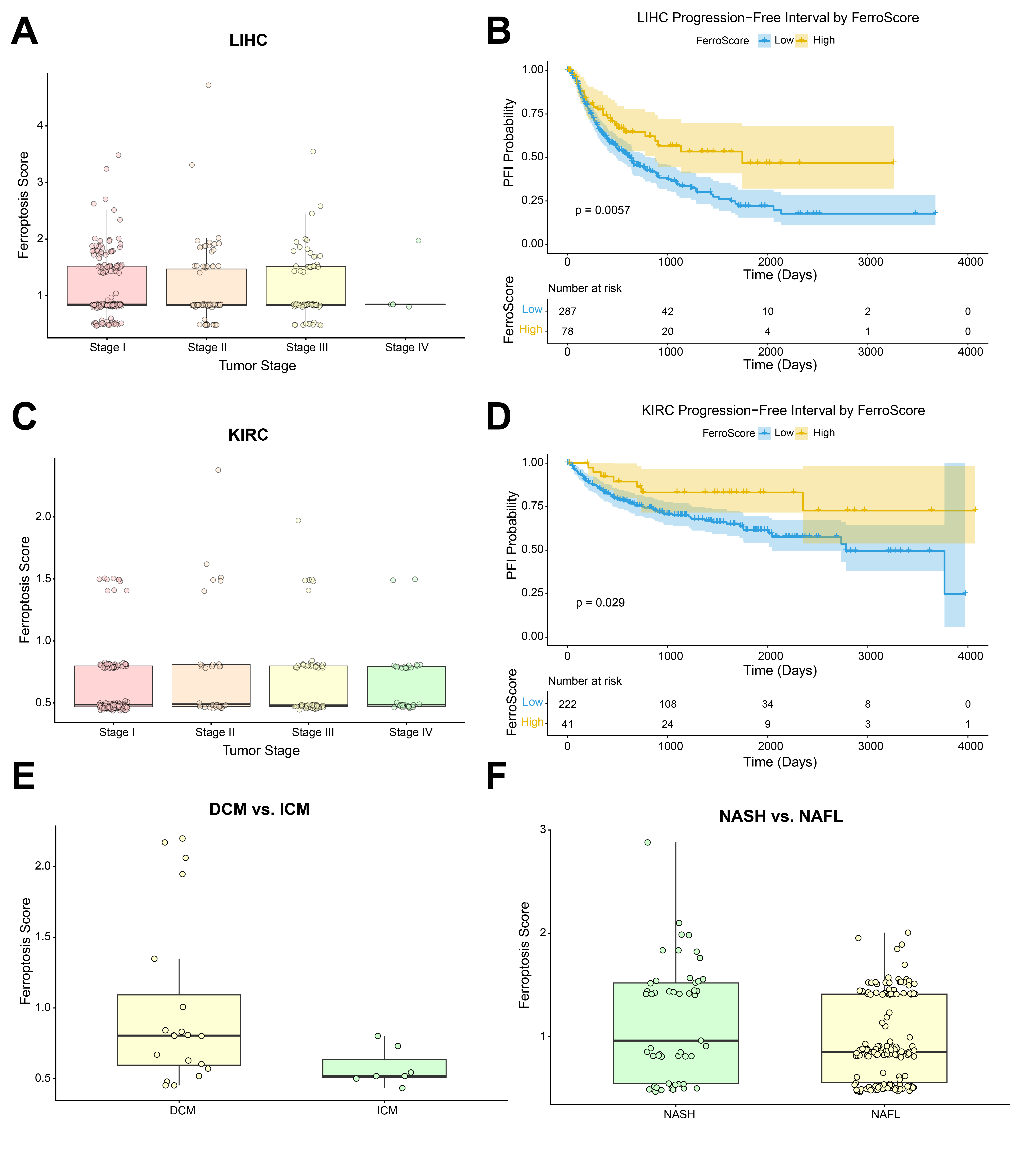


**Fig. S15. Cross-disease validation of FerroScore in oncological and non-oncological cohorts. (A–D)** Distribution of FerroScore across clinical stages and Kaplan-Meier survival curves for LIHC **(A, B)** and KIRC **(C, D)**. FerroScore acts as a favorable prognostic indicator across malignancies, likely reflecting active tumor-suppressive cell death. The non-linear stage-wise pattern seen in PAAD was less evident in LIHC and KIRC, suggesting that ferroptosis regulation is highly context-specific across different tumor ontologies. **(E)** Comparison of FerroScore between end-stage ICM and DCM. Higher scores in DCM reflect global myocardial stress, whereas lower scores in ICM likely stem from signal dilution caused by extensive fibrotic scarring in ischemic regions. **(F)** FerroScore in NAFL and NASH patients. The elevated activity in NASH aligns with ferroptosis being a key driver of hepatocyte injury and inflammation during the progression from simple steatosis to steatohepatitis.

**Supplementary Table**

**Table S1. Raw network metrics and FerroScore in MEF and A549 datasets (All values are reported with four-decimal precision).**

| **Cell Line** | **Condition** | **Q** | **D** | **k** | **BC** | **C** | **Super-node Score** | **FerroScore** |
| --- | --- | --- | --- | --- | --- | --- | --- | --- |
| MEF | AA | （Lipid）0.0119 | 2.0000 | 2 | 0 | 0 | 0.8024 | 0.8024 |
|  | Cys | （Lipid）0.0044  （GSH）0.0141 | 0.4545  0.3989 | 3  3 | 0  0 | 0  0 | 0.6918  0.6826 | 1.0135 |
|  | CysAm | （Lipid）0.0052 | 0.3015 | 2 | 0 | 0 | 0.4614 | 0.4614 |
|  | Sh-CTNS | （Lipid）0.0056  （Iron）0.0100 | 0.2553  0.2210 | 3  3 | 0  0 | 0  0 | 0.6462  0.6522 | 0.4214 |
|  | Cys+CysAm | （Lipid）0.0277 | 1.0000 | 2 | 0 | 0 | 0.6055 | 0.6055 |
|  | Cys+sh-CTNS | （GSH）0.0079  （Lipid）0.0091  （Iron）0.0097 | 1.0000  0.5714  0.5714 | 3  4  3 | 0  1  0 | 0  0  0 | 0.7162  1.1161  0.8016 | 0.9973 |
| A549 | IKE | （Lipid）-0.0063 | 1.7778 | 2 | 0 | 0 | 0.7543 | 0.7543 |
|  | RSL3 | （Lipid）0.0000 | 0.4634 | 2 | 0 | 0 | 0.4927 | 0.4927 |
|  | KO_IKE | （Lipid）0.0092  （Iron）-0.0062 | 0.1500  0.3942 | 3  3 | 0  0 | 0  0 | 0.6318  0.6776 | 0.4281 |
|  | KO_RSL3 | （Lipid）-0.0099 | 0.4956 | 2 | 0 | 0 | 0.4971 | 0.4971 |

**Table S2. Normalized network metrics and FerroScore in MEF and A549 datasets (D_norm and k_norm denote min–max normalized intra-module density and degree, respectively. All values are reported with four-decimal precision).**

| **Cell Line** | **Condition** | **Q** | **D_norm** | **k_norm** | **BC** | **C** | **Super-node Score** | **FerroScore** |
| --- | --- | --- | --- | --- | --- | --- | --- | --- |
| MEF | AA | （Lipid）0.0119 | 1.0000 | 0.0000 | 0 | 0 | 0.2024 | 0.2024 |
|  | Cys | （Lipid）0.0044  （GSH）0.0141 | 0.2273  0.1994 | 0.5000  0.5000 | 0  0 | 0  0 | 0.1463  0.1427 | 1.0254 |
|  | CysAm | （Lipid）0.0052 | 0.1508 | 0.0000 | 0 | 0 | 0.0312 | 0.0312 |
|  | Sh-CTNS | （Lipid）0.0056  （Iron）0.0100 | 0.1276  0.1105 | 0.5000  0.5000 | 0  0 | 0  0 | 0.1266  0.1241 | 0.0157 |
|  | Cys+CysAm | （Lipid）0.0277 | 0.5000 | 0.0000 | 0 | 0 | 0.1055 | 0.1055 |
|  | Cys+sh-CTNS | （GSH）0.0079  （Lipid）0.0091  （Iron）0.0097 | 0.5000  0.2857  0.2857 | 0.5000  1.0000  0.5000 | 0  1  0 | 0  0  0 | 0.2016  0.4590  0.1591 | 0.3622 |
| A549 | IKE | （Lipid）-0.0063 | 0.8889 | 0.0000 | 0 | 0 | 0.1765 | 0.1765 |
|  | RSL3 | （Lipid）0.0000 | 0.2317 | 0.0000 | 0 | 0 | 0.0463 | 0.0463 |
|  | KO_IKE | （Lipid）0.0092  （Iron）-0.0062 | 0.0750  0.1971 | 0.5000  0.5000 | 0  0 | 0  0 | 0.1168  0.1382 | 0.0161 |
|  | KO_RSL3 | （Lipid）-0.0099 | 0.2478 | 0.0000 | 0 | 0 | 0.0476 | 0.0476 |

**Table S3. Reference background sensitivity analysis of FerroScore in MEF and A549 datasets (All values are reported with four-decimal precision).**

**A. Matched control leave-one-out analysis**

| **Cell Line** | **Condition** | **Reference setting** | **DEG input** | **n_DEG** | **n_PPI nodes/edges** | **Core module(s)** | **FerroScore** |
| --- | --- | --- | --- | --- | --- | --- | --- |
| MEF | AA | NT1 reference | mRNA_NT_1 vs  mRNA_AA_1 + mRNA_AA_2 | 74 | 66/372 | No core module | NA |
|  | AA | NT2 reference | mRNA_NT_2 vs  mRNA_AA_1 + mRNA_AA_2 | 11 | 3/4 | No core module | NA |
|  | Cys | NT1 reference | mRNA_NT_1 vs  mRNA_Cys_1 + mRNA_Cys_2 | 52 | 46/254 | No core module | NA |
|  | Cys | NT2 reference | mRNA_NT_2 vs  mRNA_Cys_1 + mRNA_Cys_2 | 231 | 225/4660 | Iron | 0.4453 |
|  | CysAm | NT1 reference | NT_1 vs  Cysteamine_1 + Cysteamine_2 | 148 | 145/2110 | Lipid | 0.5051 |
|  | CysAm | NT2 reference | NT_2 vs  Cysteamine_1 + Cysteamine_2 | 127 | 121/1416 | Lipid | 0.5160 |
|  | sh-CTNS | NT1 reference | NT_1 vs  CTNS_KD_1 + CTNS_KD_2 | 320 | 315/9588 | No core module | NA |
|  | sh-CTNS | NT2 reference | NT_2 vs  CTNS_KD_1 + CTNS_KD_2 | 292 | 287/7358 | Iron | 0.4558 |
|  | Cys+CysAm | NT1 reference | DEG(Cys\|NT1) ∩  DEG(CysAm\|NT1) | 11 common (Cys 52; CysAm 148) | 9/14 | GSH | 1.2405 |
|  | Cys+CysAm | NT2 reference | DEG(Cys\|NT2) ∩  DEG(CysAm\|NT2) | 46 common (Cys 231; CysAm 127) | 35/98 | Lipid, GSH | 0.7756 |
|  | Cys+sh-CTNS | NT1 reference | DEG(Cys\|NT1) ∩  DEG(sh-CTNS\|NT1) | 20 common (Cys 52;  sh-CTNS 320) | 17/48 | Lipid, GSH | 0.7028 |
|  | Cys+sh-CTNS | NT2 reference | DEG(Cys\|NT2) ∩  DEG(sh-CTNS\|NT2) | 111 common (Cys 231;  sh-CTNS 292) | 104/580 | Iron | 0.4849 |
| A549 | IKE | DMSO1 reference | NC_DMSO_1 vs  NC_IKE_1 + NC_IKE_2 | 44 | 43/440 | Lipid, GSH | 0.9441 |
|  | IKE | DMSO2 reference | NC_DMSO_2 vs  NC_IKE_1 + NC_IKE_2 | 43 | 43/482 | Lipid | 0.7543 |
|  | RSL3 | DMSO1 reference | NC_DMSO_1 vs  NC_RSL3_1 + NC_RSL3_2 | 263 | 254/7238 | Lipid | 0.4913 |
|  | RSL3 | DMSO2 reference | NC_DMSO_2 vs  NC_RSL3_1 + NC_RSL3_2 | 260 | 251/7106 | Lipid | 0.4957 |
|  | KO_IKE | DMSO1 reference | KO_DMSO_1 vs  KO_IKE_1 + KO_IKE_2 | 45 | 43/528 | Lipid | 0.7418 |
|  | KO_IKE | DMSO2 reference | KO_DMSO_2 vs  KO_IKE_1 + KO_IKE_2 | 45 | 43/536 | Lipid, GSH | 0.8235 |
|  | KO_RSL3 | DMSO1 reference | KO_DMSO_1 vs  KO_RSL3_1 + KO_RSL3_2 | 274 | 265/8298 | Lipid | 0.4896 |
|  | KO_RSL3 | DMSO2 reference | KO_DMSO_2 vs  KO_RSL3_1 + KO_RSL3_2 | 281 | 272/8986 | Lipid | 0.4754 |

**B. Pooled control stress test**

| **Cell Line** | **Condition** | **Reference setting** | **DEG input** | **n_DEG** | **n_PPI nodes/edges** | **Core module(s)** | **FerroScore** |
| --- | --- | --- | --- | --- | --- | --- | --- |
| MEF | AA | Pooled NT reference | mRNA_NT_1 + mRNA_NT_2 +  NT_1 + NT_2 vs  mRNA_AA_1 + mRNA_AA_2 | 82 | 82/668 | No core module | NA |
|  | Cys | Pooled NT reference | mRNA_NT_1 + mRNA_NT_2 +  NT_1 + NT_2 vs  mRNA_Cys_1 + mRNA_Cys_2 | 20 | 17/74 | No core module | NA |
|  | CysAm | Pooled NT reference | mRNA_NT_1 + mRNA_NT_2 +  NT_1 + NT_2 vs  Cysteamine_1 + Cysteamine_2 | 58 | 56/356 | Lipid | 0.6011 |
|  | sh-CTNS | Pooled NT reference | mRNA_NT_1 + mRNA_NT_2 +  NT_1 + NT_2 vs  CTNS_KD_1 + CTNS_KD_2 | 91 | 89/1224 | No core module | NA |
|  | Cys+CysAm | Pooled NT reference | DEG(Cys\|pooled NT) ∩ DEG(CysAm\|pooled NT) | 3 common (Cys 20; CysAm 58) | 2/2 | No core module | NA |
|  | Cys+sh-CTNS | Pooled NT reference | DEG(Cys\|pooled NT) ∩  DEG(sh-CTNS\|pooled NT) | 1 common (Cys 20; sh-CTNS 91) | 0/0 | No core module | NA |
| A549 | IKE | Pooled DMSO reference | NC_DMSO_1 + NC_DMSO_2 +  KO_DMSO_1 + KO_DMSO_2 vs  NC_IKE_1 + NC_IKE_2 | 45 | 45/460 | Lipid,  GSH | 1.0153 |
|  | RSL3 | Pooled DMSO reference | NC_DMSO_1 + NC_DMSO_2 +  KO_DMSO_1 + KO_DMSO_2 vs  NC_RSL3_1 + NC_RSL3_2 | 268 | 258/7490 | Lipid | 0.5119 |
|  | KO_IKE | Pooled DMSO reference | NC_DMSO_1 + NC_DMSO_2 +  KO_DMSO_1 + KO_DMSO_2 vs  KO_IKE_1 + KO_IKE_2 | 48 | 45/544 | Lipid | 0.9336 |
|  | KO_RSL3 | Pooled DMSO reference | NC_DMSO_1 + NC_DMSO_2 +  KO_DMSO_1 + KO_DMSO_2 vs  KO_RSL3_1 + KO_RSL3_2 | 301 | 292/10014 | Lipid | 0.5082 |

**Table S4. Classification of signaling pathways in high-ferroptosis cell subtypes based on ferroptosis association (Literature search cut-off date: October 2025).**

| **Signaling**  **Pathways** | **Ferroptosis**  **Association** | **Specific**  **Manifestation** | **Evidence Source** | **Effect** |
| --- | --- | --- | --- | --- |
| ANGPT | Literature-confirmed correlation | Downregulated during erastin- or RSL3-induced ferroptosis | PMID：24439385 | Promote ferroptosis |
| ANGPTL | Literature-confirmed correlation | Downregulated during erastin- or RSL3-induced ferroptosis | PMID：24439385 | Promote ferroptosis |
| ANNEXIN | Literature-confirmed correlation | Knockdown of ANXA10 induces ferroptosis by inhibiting autophagy-mediated TFRC degradation | PMID：37666806 | Promote ferroptosis |
| BAG | Literature-confirmed correlation | Overexpression of Bag3 reduces the accumulation of lipid ROS and significantly inhibits the expression of SLC7A11 and SLC3A2, thereby suppressing ferroptosis | DOI:10.21203/rs.3.rs-57265/v1 | Inhibit ferroptosis |
| BMP | Literature-confirmed correlation | BMP/Smad signaling pathway affects intracellular iron levels by regulating hepcidin (iron-regulating hormone), indirectly participating in the regulation of ferroptosis | PMID：32454791 | Promote ferroptosis |
| CALCR | Literature-confirmed correlation | Calcitonin receptor (CALCR) is a gene that intersects with fibrosis and ferroptosis pathways | PMID：39611512 | Ferroptosis-related genes |
| CCL | Other | Immune response |  |  |
| COMPLEMENT | Literature-confirmed correlation | TFRC directly interacts with complement C4 to induce ferroptosis, and improving complement C3 deficiency can rescue CVB3 infection-induced ferroptosis | PMID：38169212 | Promote ferroptosis |
| CSF | Literature-confirmed correlation | LCN2 microglia induce ferroptosis in oligodendrocytes through the CSF1/CSF1R pathway | PMID：39113700 | Promote ferroptosis |
| CXCL | Literature-confirmed correlation | Downregulated after Fer-1 | PMID：25385600 | Promote ferroptosis |
| CypA | Literature-confirmed correlation | Circ_005077 enhances NADPH activity through the CyPA/p47PHOX pathway, thereby inducing ferroptosis | PMID：38622592 | Promote ferroptosis |
| EDN | Potential correlation | Interacts with PROK2 (a regulator molecule of ferroptosis) | STRING database (interacts with PROK2) |  |
| EGF | Literature-confirmed correlation | EGFR promotes MAPK, which promotes ferroptosis | PMID：28297659 | Promote ferroptosis |
| FASLG | Potential correlation | Interacts with BID (a regulator molecule of ferroptosis) | STRING database (interacts with BID) |  |
| FGF | Literature-confirmed correlation | FGF21 attenuates ferroptosis by promoting HO-1 ubiquitination and degradation | PMID：34530349 | Inhibit ferroptosis |
| GALECTIN | Literature-confirmed correlation | Tumor cells undergoing ferroptosis secrete Galectin-13. Galectin-13 binds to CD44 on the membranes of neighboring tumor cells, disrupting the interaction between CD44 and SLC7A11 on the plasma membrane, reducing the membrane localization of SLC7A11, thereby increasing the sensitivity of neighboring tumor cells to ferroptosis and triggering the spread of ferroptosis | PMID：40246981 | Spread of ferroptosis |
| GAS | Literature-confirmed correlation | GAS5 promotes the reprogramming of glucose metabolism in endothelial progenitor cells and inhibits ferroptosis | PMID：39380269 | Inhibit ferroptosis |
| GDF | Literature-confirmed correlation | GDF15 knockdown promotes erastin-induced ferroptosis by decreasing SLC7A11 expression | PMID：32209255 | Inhibit ferroptosis |
| GRN | Literature-confirmed correlation | GRN has been identified and validated as a potential ferroptosis-related biomarker in rheumatoid arthritis | PMID：37492576 | Potential ferroptosis-related biomarkers |
| IFN-II | Literature-confirmed correlation | Interferon-γ induces ferroptosis through the JAK1-2/STAT1/SLC7A11 signaling pathway; interferon-γ released by CD8 T cells downregulates the expression of SLC3A2 and SLC7A11, thereby promoting lipid peroxidation and ferroptosis | PMID：31043744；3431894；34741776；36944609 | Promote ferroptosis |
| IGF | Literature-confirmed correlation | FOXM1-activated IGF2BP3 inhibits ferroptosis by m6A-dependent regulation of RRM2 mRNA | PMID：39630361 | Inhibit ferroptosis |
| IGFBP | Literature-confirmed correlation | Overexpression of IGFBP-2 disrupts the balance of the Nrf2/SLC7A11/GPX4 ferroptosis regulatory pathway, exacerbating the progression of AD and ferroptosis | PMID：39653234 | Promote ferroptosis |
| IL16 | Other | Immune response |  |  |
| IL6 | Literature-confirmed correlation | IL6 induces lipid ROS-mediated ferroptosis; induces cell ferroptosis through the JAK2/STAT3 signaling pathway | PMID：33166496；34402724；36944609 | Promote ferroptosis |
| KLK | Potential correlation | Interacts with AR (Androgen Receptor, a regulator molecule of ferroptosis) | STRING database (interacts with AR) |  |
| LIGHT | Other | TNFSF14, immune response |  |  |
| MIF | Literature-confirmed correlation | MIF-guided small molecules enhance ferroptosis through the presumed MIF-BRCA1-RAD51 axis in homologous recombination (HR) | PMID：38924362 | Promote ferroptosis |
| MK | Other |  |  |  |
| NGF | Literature-confirmed correlation | By activating Nrf2 through the PI3K/Akt axis, NGF reduces hypoxia-induced damage in H9C2 cells, thereby inhibiting ferroptosis | PMID：39549229 | Inhibit ferroptosis |
| OSM | Potential correlation | OSM is a cytokine of the IL-6 family, interacting with IL6, JAK2, and STAT3, and also inducing ferroptosis via the JAK2/STAT3 signaling pathway | STRING database (interacts with IL6, JAK2, STAT3) | Promote ferroptosis |
| PARs | Literature-confirmed correlation | Ferroptosis is inhibited in PAR1 head and neck squamous cell carcinoma through the Rac-1 mediated JAK2/STAT3 signaling pathway | PMID：40341699 | Inhibit ferroptosis |
| PDGF | Potential correlation | Interacts with EGFR (a regulator molecule of ferroptosis) | STRING database (interacts with EGFR) |  |
| PERIOSTIN | Potential correlation | Interacts with DDR2 (a regulator molecule of ferroptosis) | STRING database (interacts with DDR2) |  |
| PLAU | Potential correlation | Interacts with CTSB (a regulator molecule of ferroptosis) | STRING database (interacts with CTSB) |  |
| PROS | Potential correlation | Interacts with SNCA (a regulator molecule of ferroptosis) | STRING database (interacts with SNCA) |  |
| SEMA3 | Other |  |  |  |
| SPP1 | Literature-confirmed correlation | Interacts with CD44 (a ferroptosis regulatory molecule); alleviates ferroptosis through the Nrf2/HO1 pathway | PMID：40321093 | Inhibit ferroptosis |
| TNF | Literature-confirmed correlation | Reducing GPX4 activity promotes ferroptosis | PMID：31160087；36944609 | Promote ferroptosis |
| TRAIL | Literature-confirmed correlation | TRAIL makes non-small cell lung cancer susceptible to ferroptosis by regulating the ASK-1/JNK1 pathway | PMID：38383815 | Promote ferroptosis |
| VEGF | Literature-confirmed correlation | Upregulated in erastin-treated samples | PMID：24844246 | Promote ferroptosis |
| VISFATIN | Literature-confirmed correlation | Inhibition of cardiomyocyte ferroptosis through the NAMPT/SIRT1 pathway | PMID：40695797 | Inhibit ferroptosis |

**References**

[1] Blondel, V. D., Guillaume, J. L., Lambiotte, R., & Lefebvre, E. (2008). Fast unfolding of communities in large networks. *Journal of statistical mechanics: theory and experiment*, *2008*(10), P10008.

[2] Blondel, V., Guillaume, J. L., & Lambiotte, R. (2024). Fast unfolding of communities in large networks: 15 years later. *Journal of Statistical Mechanics: Theory and Experiment*, *2024*(10), 10R001.

[3] Liu, Z., Zhao, Q., Zuo, Z. X., Yuan, S. Q., Yu, K., Zhang, Q., ... & Liu, Z. X. (2020). Systematic analysis of the aberrances and functional implications of ferroptosis in cancer. *Iscience*, *23*(7).

[4] He, Y., Dong, Y., Chen, Y., Zhang, G., Zhang, H., Lei, G., ... & Liu, H. (2022). Multi-omics characterization and therapeutic liability of ferroptosis in melanoma. *Signal Transduction and Targeted Therapy*, *7*(1), 268.

[5] Kim, S., Leem, G., Choi, J., Koh, Y., Lee, S., Nam, S. H., ... & Park, J. E. (2024). Integrative analysis of spatial and single-cell transcriptome data from human pancreatic cancer reveals an intermediate cancer cell population associated with poor prognosis. *Genome medicine*, *16*(1), 20.
